# Deletion of fibro-adipogenic progenitors-specific follistatin impairs muscle function and accelerates skeletal muscle atrophy in obese mice

**DOI:** 10.1186/s10020-025-01393-1

**Published:** 2025-11-21

**Authors:** Muhammad Rahil Aslam, Muhammad Bilal, Allah Nawaz, Tomonobu Kado, Shinya Abe, Nguyen Quynh Phuong, Memoona, Sana Khalid, Le Duc Anh, Ayumi Nishimura, Yoshiyuki Watanabe, Yoshiko Igarashi, Naeem Iqbal, Maki Yokoyama, Yasuhiro Onogi, Kennichi Hirabayashi, Hiroyuki Miwa, Takumi Era, Martin M. Matzuk, Seiji Yamamoto, Koichi Ikuta, Isao Usui, Kohta Kobayashi, Toshihiko Satake, Masaru Kato, Shiho Fujisaka, Kazuyuki Tobe

**Affiliations:** 1 First Department of Internal Medicine, Faculty of Medicine, University of Toyama, Toyama, 930-0194 Japan; 2 Research Center for Pre-Disease Science, Faculty of Education and Research Promotion, University of Toyama, Toyama, 930-0194 Japan; 3 Section of Integrative Physiology and Metabolism, Joslin Diabetes Center, Harvard Medical School, Boston, MA USA; 4 Laboratory of Immune Regulation, Department of Virus Research, Institute for Life and Medical Sciences, Kyoto University, Kyoto, Japan; 5 Department of Homeostatic Medicine, Medical Research Laboratory, Institute for Integrated Research, Institute of Science Tokyo, Tokyo, Japan; 6 Stem Cell Center and Tissue Bank, Phenikaa University Hospital, Phuong Canh, Hanoi, 12007 Vietnam; 7 Department of Molecular Neuroscience, Faculty of Medicine, University of Toyama, Toyama, 930-0194 Japan; 8 Faculty of Education and Research Promotion, University of Toyama, Toyama, 930-0194 Japan; 9 Rawalpindi Medical University, Rawalpindi, 46000 Pakistan; 10 Department of Diagnostic Pathology, Faculty of Medicine, University of Toyama, Toyama, 930-0194 Japan; 11 Department of Allergy and Immunology, Faculty of Medicine, Saitama Medical University, Saitama, 350-0495 Japan; 12 Department of Cell Modulation, Institute of Molecular Embryology and Genetics, Kumamoto University, 2-2-1 Honjo, Chuo-Ku, Kumamoto, 860-0811 Japan; 13 Department of Pathology and Immunology, Baylor College of Medicine, Houston, TX 77030-3411 USA; 14 Department of Pathology, Faculty of Medicine, University of Toyama, Toyama, 930-0194 Japan; 15 Department of Endocrinology and Metabolism, Dokkyo Medical University, Tochigi, Japan; 16 Department of Plastic, Reconstructive and Aesthetic Surgery, Faculty of Medicine, University of Toyama, Toyama, 930-0194 Japan; 17 Faculty of Medicine, University of Toyama, Toyama, 930-0194 Japan

**Keywords:** Fibro-adipogenic progenitors (FAPs), Follistatin, Obesity, Exercise capacity, Muscle mass

## Abstract

**Background:**

Follistatin is a potent regulator of various TGF-β superfamily members, including myostatin (MSTN) and activin A. Previous studies have shown that follistatin is crucial in enhancing myogenesis during acute muscle injury. The mechanism by which fibro-adipogenic progenitors (FAPs)-specific follistatin influences muscle homeostasis in obese mice remains unknown. Therefore, we investigated the physiological role of follistatin in PDGFRα-positive FAPs in the regulation of muscle homeostasis and exercise in obese mice.

**Methods:**

A PDGFRα-specific follistatin knockout (follistatin KO) mouse model was generated using PDGFRα-GFP-CreER^T2^ (PDGFRα-GCE) and follistatin^flox/flox^ mice. These mice were fed a 60% high-fat diet (HFD) for 20 weeks, followed by a series of analyses, including exercise tolerance test, grip strength test, glucose and insulin tolerance assays, gene expression analysis, histology, western blotting, and immunohistochemistry.

**Results:**

We showed that follistatin KO mice had reduced expression of *Fst* in skeletal muscle and white adipose tissue. We also showed that follistatin KO mice exhibited decreased exercise performance and altered skeletal homeostasis during obesity. Deletion of follistatin in FAPs activated the MSTN: Activin A/SMADs signaling pathways, which negatively impacted muscle homeostasis. Furthermore, follistatin KO mice showed reduced muscle mass, increased muscle degradation, and atrophic myofibers. Mitochondrial biogenesis, oxidative phosphorylation, and fatty acid oxidation were also altered in the skeletal muscles of follistatin KO mice.

**Conclusion:**

Follistatin plays a protective role in mice by maintaining the metabolic health of skeletal muscles; it restores muscle function during HFD challenge, thereby reducing diet-induced obesity-related complications.

**Graphical Abstract:**

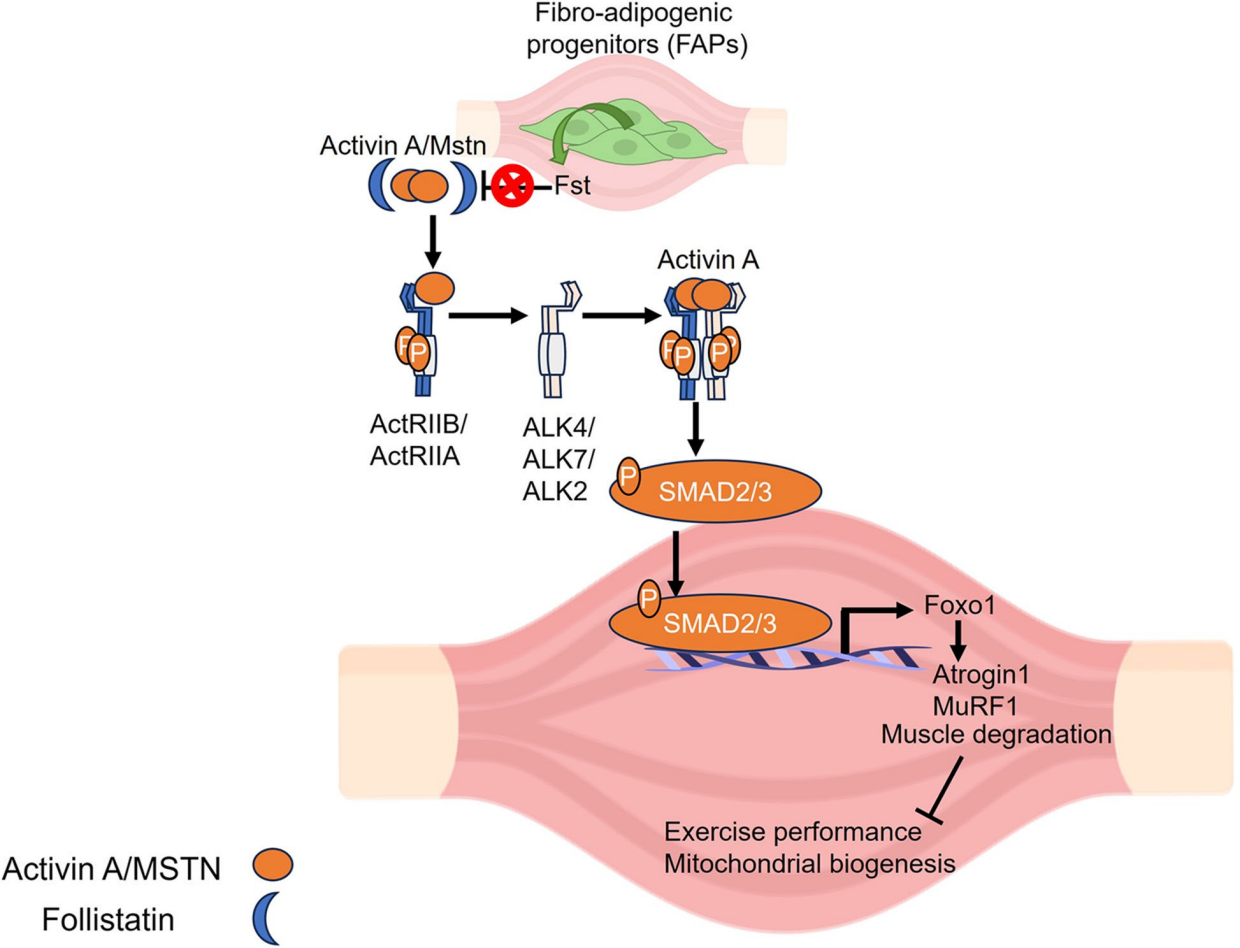

**Supplementary Information:**

The online version contains supplementary material available at 10.1186/s10020-025-01393-1.

## Background

Skeletal muscles are clinically significant organs with excellent regenerative potential, which play important roles in cases of muscle damage and musculoskeletal disorders (Kendal, et al. 2019). Muscle weakness is a typical indication of muscle degradation and is characterized by reduced physical performance and muscular atrophy (Larson and Wilbur 2020). Approximately 40% of the total body mass in humans is composed of skeletal muscles, which contribute to glucose uptake (nearly 80%) after meals or upon insulin stimulation (Sylow et al. 2021). The mechanism underlying muscle weakness is complicated and is directly associated with an imbalance in the muscle microenvironment, which consists of diverse cell populations. Unbiased clustering using single-nuclei RNA sequencing revealed all the main cell types residing in skeletal muscles, including myonuclei, smooth muscle cells, endothelial cells, satellite cells, fibro-adipogenic progenitors (FAPs), and immune cells (Petrany et al. 2020).

Several studies have demonstrated the crucial role of FAPs in maintaining muscle homeostasis and its regenerative potential. FAPs promote skeletal muscle regeneration following injury (Joe et al. 2010; Uezumi et al. 2011; Wosczyna et al. 2019; Lukjanenko et al. 2019; Nawaz et al. 2022). Being pluripotent progenitor cells, FAPs have the ability to differentiate into various cell lineages, including fibroblasts, adipocytes, osteoblast, and possibly chondrocytes (Joe et al. 2010; Uezumi et al. 2010; Lees-Shepard et al. 2018). FAPs are involved in maintaining the muscle stem cell pool and indirectly maintaining skeletal muscle mass. However, FAP deletion results in muscle stem cell dysfunction and reduced skeletal muscle mass (Wosczyna et al. 2019). Thus, FAPs play key roles in the regulation of skeletal muscle growth and homeostasis. PDGFRα is used as a universal marker to identify FAPs in humans and mice (Joe et al. 2010; Uezumi et al. 2010; Contreras et al. 2021). Recently, we reported that FAPs secrete follistatin (FST), a promyogenic factor actively involved in promoting muscle regeneration following injury (Nawaz et al. 2022). However, the mechanism via which FAPs-derived follistatin regulates exercise capacity and skeletal muscle homeostasis in obesity remains to be explored.

Obesity has become a leading pandemic, and more than 44% of adults worldwide are predicted to be overweight (Stevens et al. 2015; Blüher 2019). Obesity primarily targets skeletal muscles, white adipose tissue (WAT), and the liver and induces insulin resistance, leading to the development of type 2 diabetes.

Follistatin is associated with several metabolic diseases (Hansen et al. 2013). Owing to its para- and auto-crine effects, follistatin binds to and antagonizes various TGF-β family members, including myostatin (MSTN) and activin A, thereby neutralizing their activity (Jones et al. 2007). MSTN is a potent negative regulator of muscle mass and myofiber size, affecting muscle growth and development. Deletion of *Mstn* results in an increment in skeletal muscle mass, producing a phenotype, “double-muscle”, across various animal species and humans (Kambadur et al. 1997; Grisolia et al. 2009; Dilger et al. 2010). Further studies revealed that MSTN partially negatively regulates muscle mass, which also cooperates with activin A (Lee et al. 2020).

Both MSTN and activin A share common receptors that transmit their signals by binding to two types of transmembrane serine/threonine kinase receptors: activin receptor type II (ACTRIIA and ACTRIIB) and type I receptors (activin-like kinase (ALK)2, 4, 5, 7). (Pangas and Woodruff 2000; Namwanje and Brown 2016). Recruitment of the type I receptors occurs after the binding of these ligands to the type II receptors, which leads to the phosphorylation and subsequent activation of kinase activity (Namwanje and Brown 2016). These type I activin-phosphorylated receptors further phosphorylate intracellular mothers against the decapentaplegic homolog (SMAD) 2/3, which forms a complex with SMAD4, translocates into the cell nucleus, and activates or represses its target genes, thereby regulating muscle homeostasis (Lee et al. 2020).

Therefore, in this study, we investigated the role of FAPs-derived follistatin in the skeletal muscles of high-fat diet (HFD)-fed obese mice. Our findings revealed that deletion of FAPs-specific follistatin decreased exercise capacity and altered skeletal homeostasis in mice. Additionally, it resulted in the upregulation of the MSTN:activin A/SMAD signaling cascade, thereby regulating muscle homeostasis. Further analysis revealed that the FAPs-specific follistatin (follistatin KO) mice had reduced muscle mass, increased muscle degradation, and atrophic myofibers. We also observed mitochondrial dysfunctions and altered fatty acid oxidation in muscle of follistatin KO mice. Collectively, these findings indicate that follistatin plays a protective role in mice by maintaining the metabolic health of skeletal muscles and restoring muscle function during HFD challenge, thereby reducing diet-induced obesity-related complications such as sarcopenia.

## Materials and methods

### Animals

Transgenic PDGFRα-GFP-CreER^T2^ (PDGFRα-GCE) × Follistatin ^flox/flox^ (Follistatin^f/f^) mice were used in the experiments and genotyped as described previously (Nawaz et al. 2022). Follistatin KO mice and their littermate control, follistatin^f/f^ mice (six weeks old), were used in various experiments. All animals were maintained under a 12 h light-12 h dark cycle at 22 °C and 45 ± 5% humidity. All animals were allowed access to normal chow (NC) (CE-2; CLEA, Japan) or HFD (60% calorie fat, Research Diets, Japan) and water ad libitum. At the age of 6 weeks, the mice were fed HFD for 20 weeks. The C57BL/6 J mice at the age of 6 weeks were fed an HFD for 12 and 24 weeks along with their NC control group. The animals were transferred to clean cages once a week. All animal experiments conducted in this study adhere to the ARRIVE guidelines. All animal care and experimental protocols were approved by the Animal Experiment Committee of the University of Toyama, Toyama, Japan (authorization number A2021MED-17).

### Genotyping

Genomic DNA was extracted from mouse tail using DirectPCR® lysis reagent (Viagen, Los Angeles, CA, USA) following the manufacturer’s guidelines. DNA was genotyped using polymerase chain reaction (PCR) with the Tks Gflex DNA polymerase kit (TaKaRa, Shiga, Japan), following the manufacturer’s guidelines. The PCR conditions were as follows: one cycle of 95 °C for 5 min, 30 cycles of 94 °C for 60 s, 58 °C for 60 s, 72 °C for 90 s, and one cycle of 72 °C for 10 min, followed by incubation at 4 °C for indefinite time. The sequences of the primers from Invitrogen™ Life Technologies, Tokyo, Japan, used for genotyping of follistatin^f/f^ mice were as follows: Fst primer 1 (forward) 5'-CCTCCTGCTGCTGCTACTCT-3, Fst primer 2 (reverse), 5'-AGCATCCGCTAAGCGTAAAA-3'. The expected product sizes were 550 bp (floxed mice) and 500 bp (wild type (WT) mice). The PCR conditions for the PDGFRα-GCE mice were as follows: one cycle of 94 °C for 2 min, 30 cycles of 98 °C for 10 s, 60 °C for 30 s, one cycle of 68 °C for 1 min, 72 °C for 2 min, and finally incubation at 4 °C for indefinite time. The sequences of the primers used for genotyping of PDGFRα-GCE mice were as follows: 5ʹ-WT: AAGACGATTCACACTGCCGATG; 3ʹ-WT: AGACAGCTGAGGACCAGAAAGA; 3ʹ KI: TGGTGCAGATGAACTTCAGGGT. The PCR products were separated by electrophoresing on a 1.5% agarose gel for 30 min. Ethidium bromide (1:1000) was added to the agarose gel to visualize the products. All the reagents used in this study are provided in the Supplementary Table 1 [See additional file 2].

### Tamoxifen (TAM) preparation and administration

TAM was purchased from Sigma-Aldrich and was dissolved in sunflower oil (Fujifilm WAKO, Japan). TAM (150 mg/kg body weight (BW)) was orally administered to follistatin KO and control littermates via gavage starting after 16 weeks of HFD-feeding for five consecutive days. One week after the recovery period, the mice were subjected to exercise tolerance and grip strength tests.

### Exercise tolerance test (ETT)

Mice were subjected to ETT as reported previously (Bilal et al. 2025). Shortly before the commencement of the actual test, firstly, the mice were trained for one day. The mice were allowed to run on a treadmill equipped with a shock grid connected to the rear end for 10 min at a 10-m/min speed. On the actual day of the test, access to food was restricted for 2 h while access to water was allowed. After 5 min, the total distance covered by the mice and the number of shocks at a specific time were recorded. Mice were considered to be exhausted if they could not run further and stayed on the grid for up to 15 s, followed by their removal from the treadmill. Observations were made every 5 min until the mice were exhausted. The distance covered till exhaustion set in was determined as follows:

$$\mathrm S\left(\mathrm{distance}\;\mathrm{in}\;\mathrm{meters}\right)=\mathrm V\left(\mathrm{speed}\;\mathrm{in}\;\mathrm{meters}/\min\right)\times\mathrm T\left(\mathrm{time}\;\mathrm{in}\;\mathrm{minutes}\right),$$

### Grip strength test (GST)

GST was performed by determining mice hanging time (Bilal et al. 2025). A stand was prepared manually using a net, and the mice were allowed to hang while holding the net. Before the actual test, the first group of mice was trained for one day. On the test day, access to food was restricted for 2 h. The mice were allowed to hang repeatedly from a net, followed by dropping, and observations were made. The maximum hanging time and number of droppings in 5 min were recorded.

### Glucose tolerance test (GTT) and intraperitoneal insulin tolerance test (Ip-ITT)

To perform the intraperitoneal glucose tolerance test (Ip-GTT), follistatin KO and follistatin^f/f^ mice, either fed normal chow (NC) or HFD for 19–20 weeks were fasted for 4 h and injected with glucose (1 mg/g BW) intraperitoneally. The chow Blood samples were collected from the tail vein at various time points over 2 h (0, 30, 60, 90, and 120 min). For Ip-ITT, the mice were fasted for 4 h, followed by intraperitoneal administration of human insulin (NC 0.8 U/kg BW, HFD 1.2 U/kg BW). Blood samples were collected from the tail vein at various time points for over 2 h (0, 15, 30, 45, 60, 90, and 120 min). StatStrip® Express 900 (Nova Biomedical, Waltham, MA, USA) strips were used to measure blood glucose levels.

### Flow cytometry analysis

We performed flow cytometry analysis of gastrocnemius muscle (GC) muscle from the follistatin KO mice and control mice as described previously (Nawaz et al. 2022) and investigated FAPs (+) cell population. In brief, the GC muscle was harvested and digested by using collagenase II (2 mg/mL) and dispase (2 mg/mL) in KRHAG buffer for 45 min at 37 °C in a water bath under agitation. After that, the mixture was passed through the 18-gauge syringe and incubated as above for 10 min. The digestive tissue mixture was passed through 70 μm cell strainer and centrifuged at 1500 RPM for 5 min at 4 °C. The pellet was washed again with KRHAG buffer, followed by lysis in 1 × lysis buffer for 10 min at room temperature. The cell suspension was then centrifuged as above and washed with fluorescence activated cell sorting (FACS) buffer. The single-cell suspension was FC blocked at room temperature for 10 min, followed by staining with flow cytometry antibodies for CD45, CD31, SCA-1, and PDGFRα for 30 min on ice (Antibody details are given in Supplementary Table 1). The cell suspension was washed twice with FACS buffer and then subjected to flow cytometric analysis. The 7AAD- cell population was further gated as lineage negative (CD45-;CD31-), and PDGFRα +; SCA1 + cells (FAPs (+)) and PDGFRα-;SCA1- cells (FAPs (-)) cell populations were analyzed and FACS was performed. The BD FACS ARIA™-II machine and Flowjo offline software were used for the analysis.

### Immunohistochemistry (IHC)

Tissues were embedded in paraffin, and 5–10 μm-thick sections were obtained for hematoxylin and eosin (H & E) staining as described previously (Bilal et al. 2021). The images were captured using a Keyence BZ-X800 fluorescence microscope (Osaka, Japan).

Frozen sections were prepared as described previously (Nawaz et al. 2022; Nawaz et al. 2017). Primary and secondary antibodies were used according to the manufacturer’s instructions. The primary antibodies used were against p-SMAD2/3 (1:100; Cell Signaling Technology, Danvers, Massachusetts, USA), anti-Laminin alpha2 (1:100; Santa Cruz), and p27 (1:100; Cell Signaling Technology). The relevant secondary antibodies were used at a 1:250 dilution, and a 1:400 dilution of 4',6-diamidino-2-phenylindole (DAPI) was used. The images were captured using an LSM900 confocal microscope (ZEISS, Germany).

### Western blotting

Tissues were harvested and immediately snap frozen in liquid nitrogen, followed by storage at −80 °C. Western blotting was performed using a previously described method with minor modifications (Nishida et al. 2020). Briefly, the tissues were homogenized in lysis buffer consisting of 10 mM EDTA, 25 mM Tris–HCl (pH 7.4), 50 mM Na_4_P2O_7_, 10 mM Na_3_VO_4_, 100 mM NaF, 2 mM phenylmethylsulfonylfluoride, and 1% Nonidet P-40 using a Multi-Beads Shocker cell disrupter (Yasui Kikai Corporation, Osaka, Japan). After lysis, the crude lysates were centrifuged at full speed to remove the cell debris or insoluble material. Protein (2 μg/μL) was mixed with the loading buffer and denatured for 5 min at 95 °C. The denatured protein lysates were then run on a 4 − 12% sodium dodecyl sulfate polyacrylamide gel and transferred to a nitrocellulose membrane (Immobilon-P, Millipore, Billerica, MA, USA). The membrane was blocked with 5% skim milk in Tris-buffered saline-Tween 20 (TBS-T) for 1 h, followed by three washes with TBS-T. The membranes were then stained with anti-GDF8 (1:300, Bioss, USA) and FoxO1, α-Tubulin, p-AMPKα, AMPKα (1:1000, Cell Signaling Technology), and PGC1α (1:500, Santa Cruz) overnight at 4 °C. The membranes were washed again and incubated with a secondary antibody (1:2000) at room temperature for 2 h, followed by washing with TBS-T. Before detection, the membranes were rinsed with enhanced chemiluminescence prime detection reagent and images were obtained using a Bio-Rad ChemiDoc Touch™ (California, USA).

### Measurement of myofiber size

The size of the myofibers was determined using the ‘Set Scale’ function in ImageJ 1.53a (National Institute of Health, Bethesda, MD, USA) software from randomly selected 20 × fields/specimen. Six random fields for each depot, slide, and specimen were analyzed.

### Real-time PCR (RT-PCR)

RT-qPCR was performed as described previously (Watanabe, et al. 2023; Igarashi et al. 2018). Briefly, RNA was extracted from the tibialis anterior (TA) tissues using a Qiagen RNeasy kit. Reverse transcription was performed using the PrimeScript RNA kit (TaKaRa, Shiga, Japan) according to the manufacturer’s instructions. RT-PCR was performed using gene-specific primers and TB Green® FastqPCR master mix (Tli RNaseH Plus; TaKaRa, Shiga, Japan) following the manufacturer’s guidelines. The relative mRNA expression levels were calculated using the ΔΔCt method. *Tf2b* or *Actb* was used as a control to normalize the mRNA levels. The SYBR Green primer sequences used in this study are listed in Supplementary Table 2.

### *In-vitro* and *ex-vivo* co-culture

All the *in-vitro* and *ex-vivo* experiments were conducted as described previously, with little modifications (Nawaz et al. 2022). In brief, C2C12 cells were grown in growth medium containing high glucose Dulbecco’s modified Eagle’s medium (DMEM, Thermo Fisher Scientific) supplemented with 10% fetal bovine serum (FBS) (Gibco™ 10,437–028) with antibiotics at 37 °C in 5% CO2. TA and GC muscles were harvested and digested as described in flow cytometric sample preparations. For the co-culture of FAPs with C2C12, first, we isolated the FAPs as described above by FACS in 20% FBS in DMEM. Gelatin coating is a widely used method for culturing FACS or MACS-sorting of FAPs (Lukjanenko et al. 2019; Gao et al. 2025). We coated the plates with 0.1% gelatin solution in PBS (-) for 1 h at 37 °C. Gelatin solution was removed, and after 10 min wait at room temperature, the isolated FAPs were then grown in gelatin-coated plates. After 24 h, non-adherent cells were removed and the media were changed to fresh growth media after one wash with PBS (-). The adherent FAPs were maintained in 10–20% FBS in DMEM. 30,000 C2C12 cells were cultured in 12-well plates, and after reaching confluency of 70–80%, the growth media were removed, and 10,000 FAPs were added in each well along with the differentiation medium (3–4% horse serum). After 48 h and day 5, cells were harvested, and RNA was extracted. For conditional medium (CM) experiments, the growth media were removed and washed with PBS once, and 0.1% FBS in DMEM was added to the FAPs cultured plates. CM was collected after 24 h. CM was passed through a 0.22 μm filter and stored at −80 °C for later use. C2C12 cells were cultured as described above and treated with CM. At day 5, cells were harvested, and RNA was extracted. The gene expression analysis was performed by using RT-PCR.

### Statistical analysis

Statistical analyses were performed using the GraphPad Prism 9 software (ver. 9.1.2; GraphPad Software, San Diego, CA, USA). Data are shown as means ± standard error of the mean (SEM). Statistical significance was set at *p* < 0.05 and measured using the unpaired Student *t*-test and one-way or two-way analysis of variance (ANOVA).

## Results

### FAPs-specific deletion of follistatin does not impact metabolic homeostasis in lean and obese mice

**Fig. 1:**
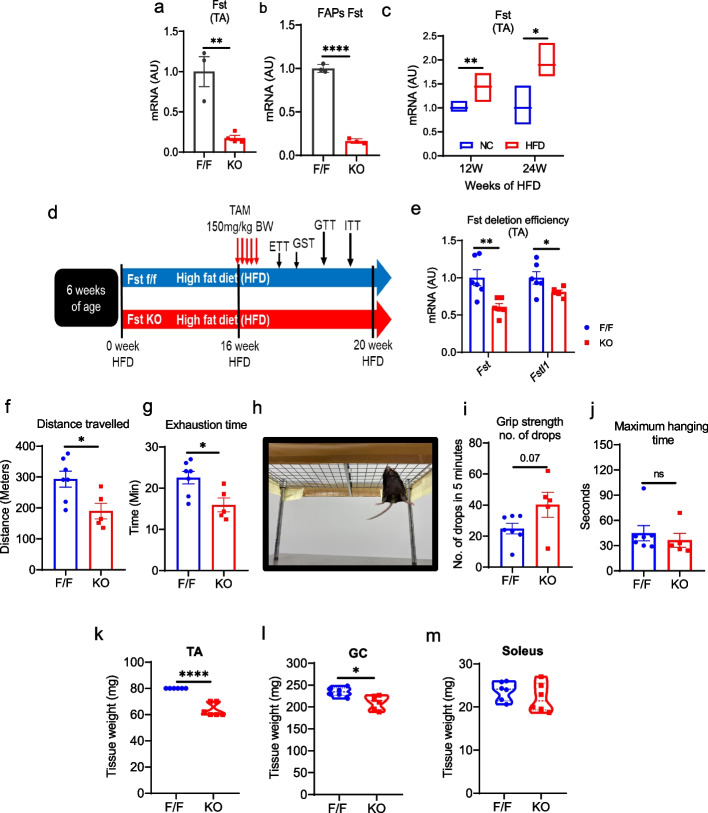
Deletion of Follistatin in Fibro-Adipogenic Progenitor Cells Altered skeletal muscle function in Obese Mice.** a** Relative mRNA expression of *Fst* in the TA of NC mice (*n* = 3, 4). **b** Relative mRNA expression of *Fst* in purified fibro-adipogenic progenitors from the TA of NC mice (*n* = 3, 3). **c** Relative mRNA expression of *Fst* in the TA of NC and HFD-fed mice (*n* = 3–5). **d** Schematic diagram showing the experimental setup. **e** Relative mRNA expression of *Fst* and *Fstl1* in the TA of obese mice (*n* = 6, 6). **f-g** Average distance traveled and time until the mice were exhausted (*n* = 7, 5). **h** Method used for grip strength test. **i** Grip strength test. Total number of drops in 5 min (*n* = 7, 5). **j** Maximum hanging time in 5-min intervals (*n* = 7, 5). **k-m** Skeletal muscle weight TA, GC, and soleus after 20 weeks of HFD feeding (*n* = 6, 6). Data are expressed as the mean ± standard error of the mean (SEM). *p < 0.05, **p < 0.01, and ****p < 0.0001, two-tailed Student *t*-tests (B-E, G, H, K-M) and the two-way ANOVA followed by Sidak’s post hoc tests (I & J). F/F Follistatin F/F mice, KO FAPs-specific follistatin KO mice, *mRNA* Messenger RNA, *AU* Arbitrary units, *TA* Tibialis anterior, *GC* Gastrocnemius

We generated FAPs-specific follistatin KO mouse model using PDGFRα-GFP-CreER^T2^ (PDGFRα-GCE) and follistatin^f/f^ mice as described previously (Nawaz et al. 2022). We conducted experiments to elucidate the physiological role of FAPs-specific follistatin deletion in skeletal muscle in lean and obese conditions. TAM was administered orally at 6 weeks of age, and mice were fed with the chow diet for a total 20 weeks. To determine the FAPs-specific Fst deletion, FAPs were FACS sorted (gating strategy is given in Supplementary Fig. S1a and S1b). Our gene expression analysis of TA muscle and FACS-sorted FAPs showed downregulation of *Fst* in the skeletal muscle of follistatin KO mice (Fig. 1a and b). There was no difference in the body weights of follistatin KO group compared with the control group on either dietary intervention (Supplementary Fig. S2a, d). We also did not observe any difference in glucose tolerance and insulin tolerance in follistatin KO mice compared with the control group in NC (Supplementary Fig. S2b and 2c) and HFD-fed mice (Supplementary Fig. S2e and g), suggesting that deletion of follistatin in FAPs does not alter glucose metabolism.

### Deletion of FAPs-specific follistatin impaired skeletal muscle function in obese mice

To assess the impact of FAPs-specific deletion of follistatin under obesity-induced metabolic stress, first, we used 12-week and 24-week HFD-fed C57BL/6 J mice to examine whether the *Fst* expression alters with obesity. We found increased expression of *Fst* in the HFD-fed mice skeletal muscle (Fig. 1c), which might be to maintain the skeletal muscle homeostasis during obesity. Therefore, to investigate the effect of obesity on skeletal muscle and glucose homeostasis of follistatin KO mice. Following oral administration of TAM as shown in the experimental protocol (Fig. 1d), the BW of the two phenotypes was comparable (Supplementary Fig. S3a). Gene expression analysis confirmed that *Fst* was successfully downregulated in the TA (Fig. 1e) and epididymal white adipose tissue (eWAT) (Supplementary Fig. S2b) of follistatin KO mice compared to that in follistatin^f/f^ mice. The expression of *Fstl1*, another gene of the follistatin family, structurally similar to follistatin, might have a supportive role during myogenesis, and its expression was also downregulated in follistatin KO mice (Fig. 1e).

Primarily, it implies that since follistatin is a promyogenic factor, its role in exercise has not been established. Therefore, we presumed that deletion of follistatin might alter exercise performance in follistatin KO mice. To test this hypothesis, we performed an exercise tolerance test (ETT). Mice were allowed to run on a treadmill until exhaustion set in. We showed that follistatin KO mice exhibited reduced exercise performance and were exhausted. The distance traveled (Fig. 1f) and exhaustion time (Fig. 1g) were reduced in follistatin KO mice. These follistatin KO mice showed increased number of droppings in the grip strength evaluated by hanging (Figs. 1h and i), whereas the maximum hanging time was comparable (Fig. 1j). These data suggested that FAPs-derived follistatin deletion may have a moderate effect on grip strength in mice.

Interestingly, we found a massive reduction in skeletal muscle mass, especially TA (Fig. 1k) and GC mass (Fig. 1l) in the follistatin KO mice compared to that in the control follistatin^f/f^ mice. However, the soleus muscle weight did not change (Fig. 1m). Additionally, the weights of eWAT, inguinal white adipose tissue (iWAT), brown adipose tissue (BAT), and liver did not change (Supplementary Fig. S3c-f). Taken together, these results indicated that follistatin deletion in FAPs does not influence glucose metabolism in NC as well as HFD-fed mice, but rather plays an important role in skeletal muscle homeostasis, not only in acute muscle injury (Nawaz et al. 2022) but also during physical activity and HFD challenge.

### Effects of FAPs-specific deletion of follistatin on TGF-β signaling and cellular senescence

**Fig. 2:**
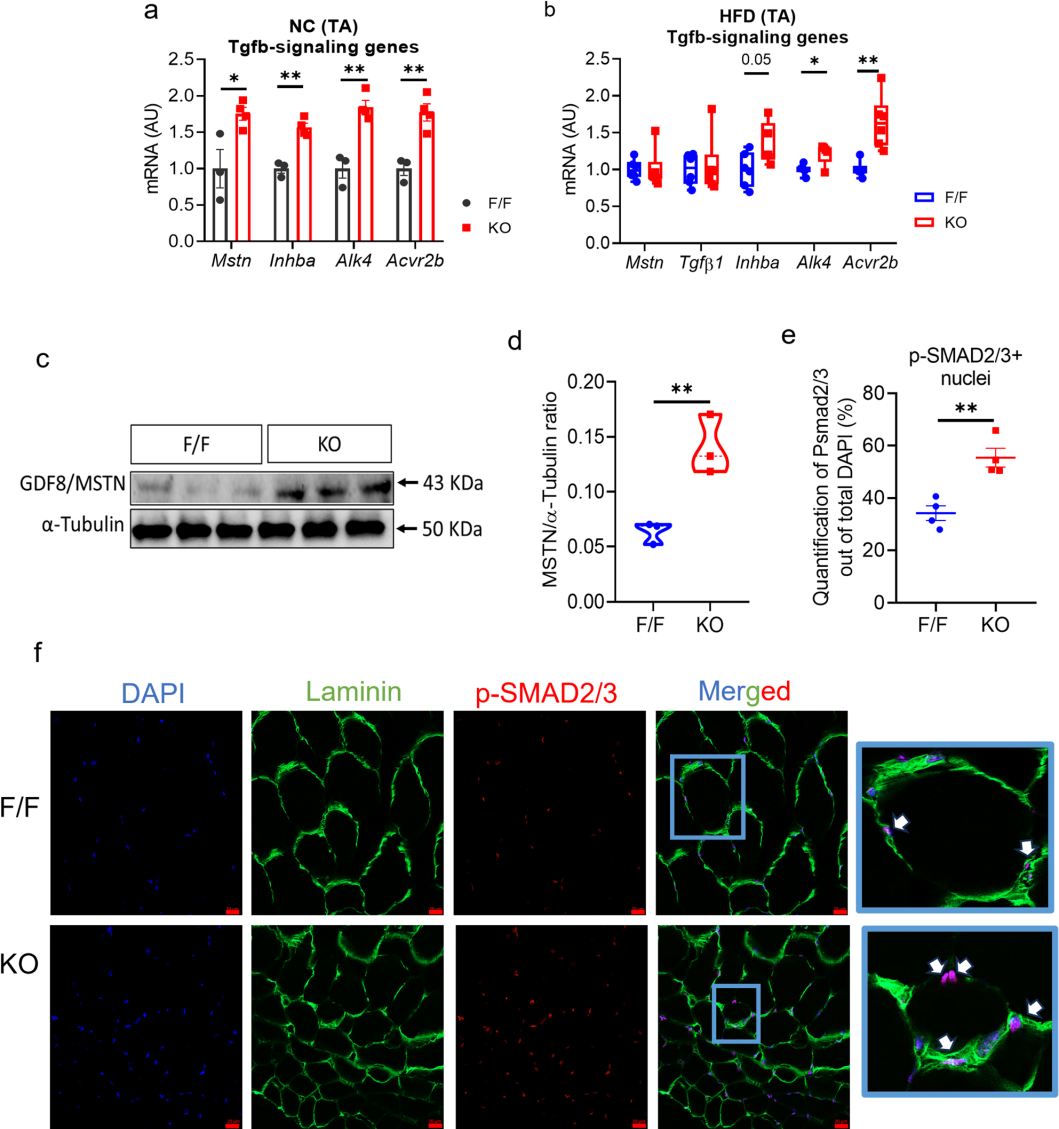
Deletion of Follistatin in FAPs Enhanced Activin A and MSTN Signaling and Activated pSMAD2/3 Pathway. **a** Relative mRNA expression of TGF-β signaling genes *Mstn* and *Inhba* (Activin A) genes and their receptors *Alk4* and *Acvr2b* in the TA tissue of lean mice (*n* = 3, 4). **b** Relative mRNA expression of *Mstn, Tgfb1, Inhba, Alk4*, and *Acvr2b* in the TA tissue of obese mice (*n* = 6, 5-6). **c** Representative western blot images. **d** Quantification of GDF8/MSTN protein levels from TA tissue of obese mice (*n* = 3, 3). **e** Quantification of p-SMAD2/3+ nuclei out of total DAPI-stained nuclei in the TA from obese mice (*n* = 4 each). **f** Representative confocal images of TA stained with anti-p-SMAD2/3 (red), anti-laminin (green), and DAPI (blue) in obese mice (*n* = 4 mice/group/experiment; scale bar, 20 µm). Data are expressed as the mean ± standard error of the mean (SEM). *p < 0.05, and **p < 0.01, two-tailed Student t-tests (B-D). F/F Follistatin F/F mice, KO FAPs-specific follistatin KO mice, *mRNA *messenger RNA, *AU,* arbitrary units; *TA,* Tibialis anterior

MSTN and activin A are the predominant negative regulators of TGF-β family in skeletal muscle, and follistatin antagonizes both. Deletion of follistatin in FAPs might increase MSTN and activin A signaling. Previous studies have reported that MSTN and activin A share common type I and II receptors that transmit their signals. Initially, our gene expression analysis from the TA muscle of NC-fed mice showed increased expression of *Mstn* as well as *Inhba* gene encoding activin A (Fig. 2a). Their subsequent binding receptors, such as type I *Alk4* and type II receptor *Acvr2b,* were also upregulated in the muscle of follistatin KO mice as compared to control mice (Fig. 2a). Likewise, in the TA muscle of HFD mice, we determined the expression of *Mstn*, *Tgf-β1* and activin A-coding gene, *Inhba*. The expression of *Mstn, Tgf-β1* was unchanged; however, the expression of *Inhba* along with their subsequent receptors* Alk4* and *Acvr2b* was also enhanced in follistatin KO mice (Fig. 2b). Further, our western blot analysis showed an increased level of MSTN protein in the TA of follistatin KO mice under HFD-fed conditions (Fig. 2c, d). We also examined the MSTN by IHC. Staining with MSTN antibody also revealed increased MSTN-positive nuclei (out of total DAPI^+^ nuclei) (Supplementary Fig. S4a, b). These findings further strengthened our hypothesis that the reduction in skeletal muscle mass was indeed due to increased MSTN and activin A signaling, which lead to the SMAD2/3 phosphorylation. Next, we examined *p*-SMAD2/3 by IHC. Staining with a *p*-SMAD2/3 and Laminin antibodies revealed that the proportion of *p*-SMAD2/3^+^ DAPI^+^ nuclei (out of total DAPI^+^ nuclei) was enhanced in the TA of follistatin KO mice compared to that in the control mice (Fig. 2e, f). The increase in *p*-SMAD2/3 signal was observed in nuclei inside the muscle fiber, but also in the other cell types. Taken together, these data suggested that deletion of FAPs-derived follistatin enhanced MSTN/activin A/*p*-SMAD2/3 pathway, thereby accelerating skeletal muscle dysfunction in obesity.

**Fig. 3:**
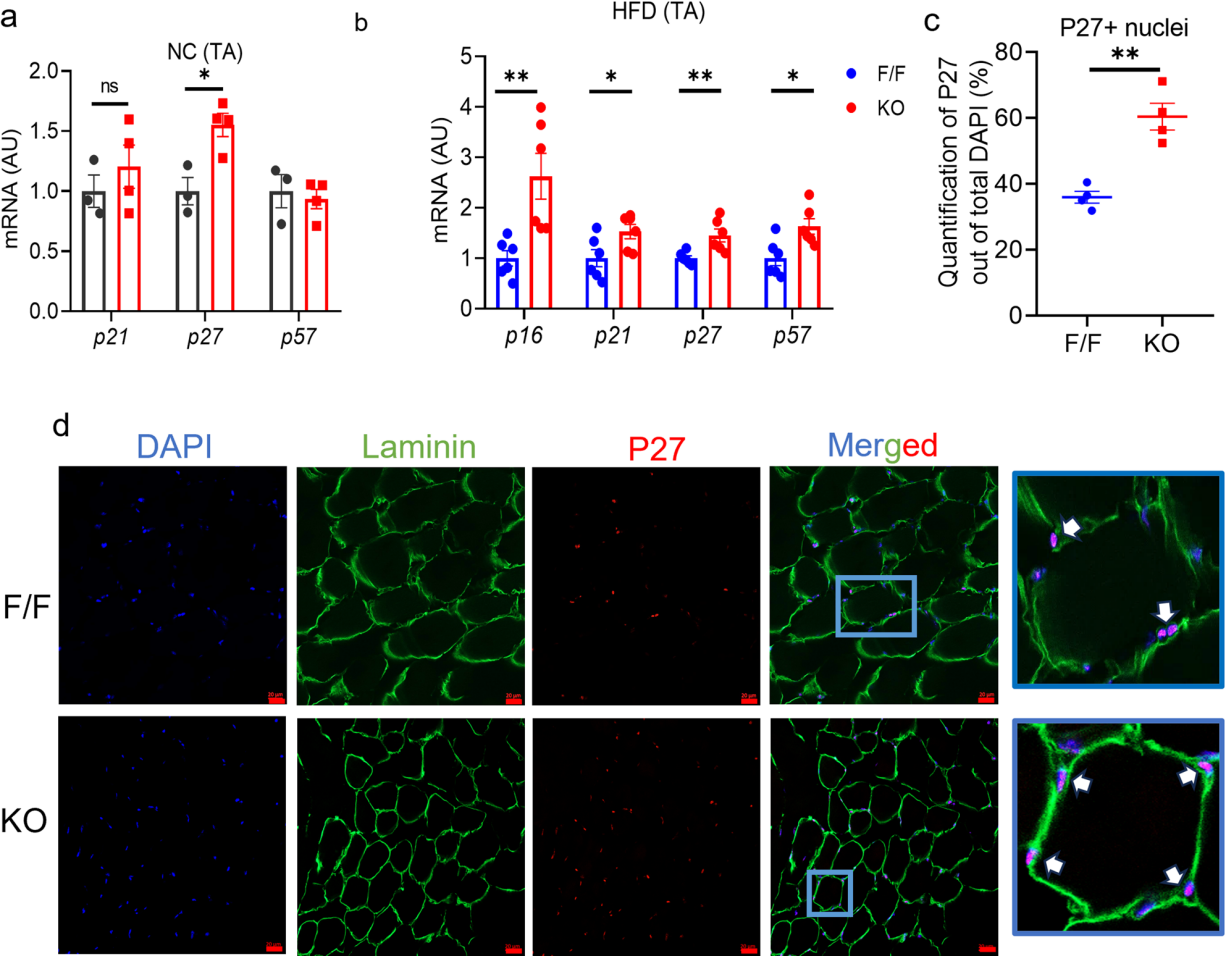
Deletion of Follistatin in FAPs Activated the TGF-β1 Downstream Signaling Pathway. **a** Relative mRNA expression of *p21, p27,* and* p57* genes in the TA tissue of lean mice (*n* = 3, 4). **b** Relative mRNA expression of *p16, p21, p27,* and* p57* genes in the TA tissue of obese mice (*n* = 6, 6). **c** Quantification of p27^+^ nuclei out of total DAPI-stained nuclei in the TA paraffin sections from obese mice (*n* = 4 each). **d** Representative confocal images of TA frozen sections stained with anti-p27 (red), anti-laminin (green), and DAPI (blue) in obese mice (*n* = 4 mice/group/experiment; scale bar, 20 µm). Data are expressed as the mean ± standard error of the mean (SEM). *p < 0.05, **p < 0.01 and ***p < 0.001, two-tailed Student *t*-tests (A, B). F/F Follistatin F/F mice, KO FAPs-specific follistatin KO mice, *mRNA* Messenger RNA, *AU* Arbitrary units, *TA* Tibialis anterior

We next focused on the role of the downstream signaling members of TGF-β, including *p16, p21, p27*, and *p57,* which are involved in the regulation of cell cycle arrest. A previous report has shown that MSTN levels were low in the skeletal muscles of mice lacking *p27*. Here, we found increased MSTN signaling in follistatin KO mice, which may reduce muscle mass. TGF-β downstream signaling might increase in follistatin KO mice. Initially, we observed only increased *p27* expression in the TA muscle of NC fed follistatin KO mice (Fig. 3a). Further, we performed gene expression analysis of HFD-fed mice TA muscle, which revealed that the TGF-β downstream signaling-related genes including *p16, p21, p27*, and *p57* were upregulated in follistatin KO mice (Fig. 3b). Consistent with this, immunofluorescence analysis (staining with anti-p27^Kip^ and anti-laminin antibodies) confirmed that the proportion of p27^+^ DAPI^+^ nuclei was enhanced in the TA muscle of follistatin KO mice compared with that in follistatin^f/f^ control mice (Fig. 3c, d). The enhanced signal was observed in nuclei that were outside the muscle fiber and that might affect other cell types. These data demonstrated that *p27*, along with its other family members, may inhibit the cell cycle, and may cause cellular senescence, a feature of aging-related phenotypes.

### FAPs-specific deletion of follistatin enhanced muscle protein degradation

**Fig. 4:**
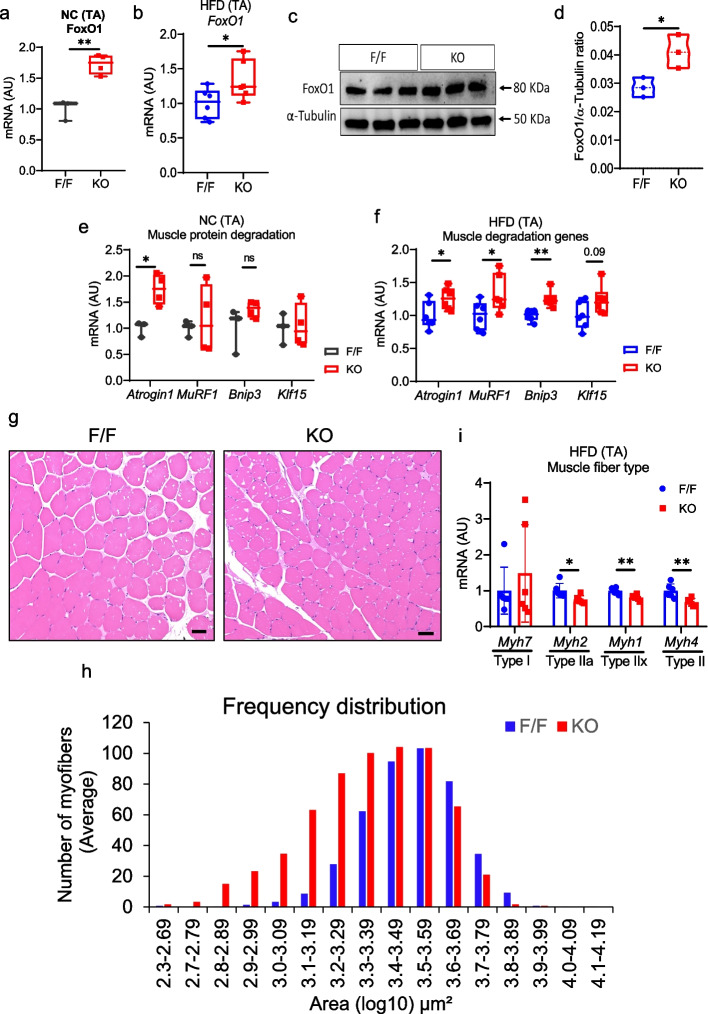
Effects of FAPs-Derived Follistatin Deletion on Muscle Protein Degradation, and Muscle Atrophy. **a** Relative mRNA expression of muscle *FoxO1* gene in the TA tissue of lean mice (*n* = 3, 4). **b** Relative mRNA expression of expression of muscle *FoxO1* gene in the TA tissue of obese mice (*n* = 6, 6). **c** Representative western blot images showing FoxO1 expression. **d** Quantification of FoxO1 protein in the TA tissue of obese mice (*n* = 3, 3). **e** Relative mRNA expression of muscle protein degradation-related genes, *Atrogin1, MuRF1, and Bnip3*, in the TA tissue of lean mice (*n* = 3, 4). **f** Relative mRNA expression of muscle protein degradation-related genes, *Atrogin1, MuRF1, Bnip3,* and *Klf15* genes in the TA tissue of obese mice (*n* = 6, 6). **g** Representative H & E staining images of TA paraffin sections (*n* = f/f mice, 4; KO mice, 3 mice/group/experiment; scale bar, 50 µm). **h** Quantification of myofiber cross-sectional area of TA (*n* = f/f mice, 4; KO mice, 3). **i** Relative mRNA expression of muscle fiber type-related genes *Myh7, Myh2, Myh1,* and *Myh4* genes in the TA tissue of obese mice (*n* = 6, 6). Data are expressed as the mean ± standard error of the mean (SEM). *p < 0.05, **p < 0.01 and ***p < 0.001, two-tailed Student *t*-tests (A, B, D, E). F/F Follistatin F/F mice, KO -FAPs-specific follistatin KO mice, *mRNA* Messenger RNA, *AU* Arbitrary units, *TA* Tibialis anterior

As shown in Fig. 1k-l, skeletal muscle mass was reduced in follistatin KO mice. We attempted to identify the molecular mechanisms underlying skeletal muscle mass loss. Recently, we reported that TGF-β1 antagonists, including follistatin, are involved in regulating myogenesis in a mouse model of acute injury (Nawaz et al. 2022). However, this remains to be elucidated in non-injured mice. Here, we performed gene expression analysis of myogenesis-related genes, including *Pax7, Myf5,* and *MyoG* in the TA muscle of lean and obese mice (Supplementary Fig. S5a-c). The expression of *Myf5* and *MyoG* was comparable in NC and HFD-fed follistatin KO mice. We observed reduced mRNA levels of Pax7 in the TA of NC mice and the GC of HFD-fed follistatin KO mice (Supplementary Fig. S5a-c). These findings reveal a selective downregulation of *Pax7*, implying differential sensitivity of myogenic regulators to the FAPs-specific follistatin KO condition. Next, we examined the effect of MSTN/activin A/*p*-SMAD2/3 signal increment on muscle protein degradation. Canonical SMAD2/3 signaling controls FOXO1 activity by promoting its dephosphorylation and nuclear translocation, thereby inducing atrophy-related genes (Goodman et al. 2013). FoxO1 is a regulator of muscle protein degradation. Gene expression analysis revealed that the *FoxO1* mRNA was upregulated in the TA of NC and HFD-fed follistatin KO mice compared to that in the control mice (Fig. 4a, b). Western blot analysis further confirmed that the FOXO1 protein level in obese follistatin KO mice was higher than that in the control littermates (Fig. 4c, d). Furthermore, we examined the expression of other muscle protein degradation-related genes such as *Fbxo32* (encoding Atrogin1)*, Trim63* (encoding MuRF1)*, Bnip3,* and *Klf15*. Only the *Atrogin1* mRNA level was increased in NC-fed follistatin KO mice. However, we found a strong impact of follistatin deletion in HFD-fed mice in which the mRNA levels of *Atrogin1, MuRF1, Bnip3,* and *Klf15* were increased in the TA of follistatin KO mice (Fig. 4e, f). Increased muscle protein degradation can lead to skeletal muscle loss and atrophy. To confirm this hypothesis, we performed H & E staining, which revealed smaller muscle fibers in the TA tissues of follistatin KO mice (Fig. 4g). Furthermore, we quantified the cross-sectional area of muscle fibers and found that the number of muscle fibers with smaller cross-sectional areas were higher in the TA of follistatin KO mice than that in the TA of control mice (Fig. 4h). Analysis of muscle fiber type by gene expression analysis showed reduced expression of type II and mixed type muscle fiber markers in the TA of follistatin KO mice as compared to control mice (Fig. 4i) which may reflect decreased fiber type maintenance under metabolic stress conditions like obesity. Collectively, these data showed that follistatin KO in skeletal muscles increased muscle protein degradation, leading to muscular atrophy and skeletal muscle loss.

### Deletion of follistatin in FAPs altered the metabolic function of skeletal muscle

**Fig. 5:**
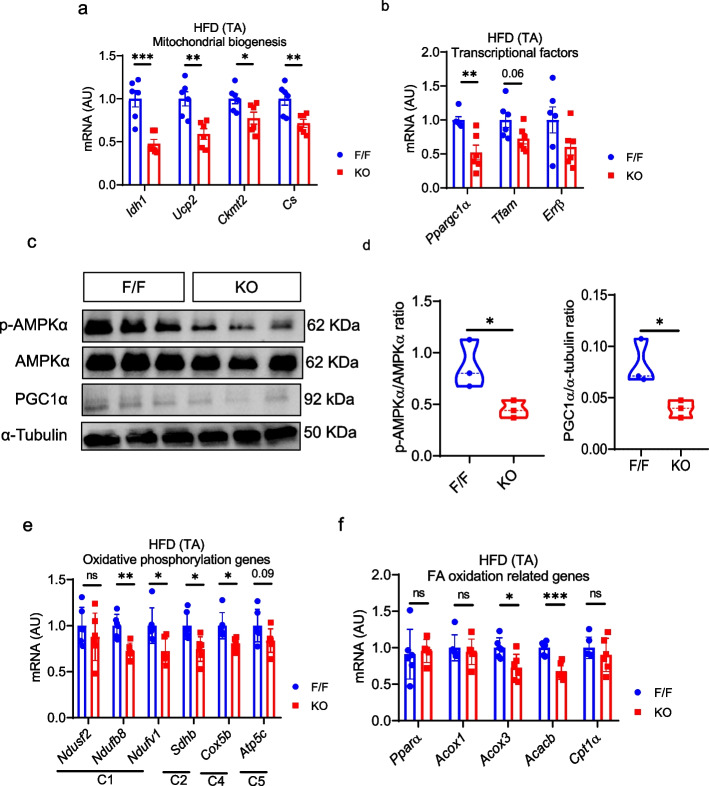
Deletion of Follistatin in FAPs Reduced Mitochondrial Biogenesis and Oxidative Metabolism. **a** Relative mRNA expression of mitochondrial biogenesis-related genes, *Idh1, Ucp2, Ckmt2* and *Cs,* in the TA tissue of obese mice (*n* = 6, 6). **b** Relative mRNA expression of transcription factors, *Ppargc1a, Tfam,* and *Errb,* which regulated mitochondrial biogenies in the TA tissue of obese mice (*n* = 6, 6). **c** Representative western blot images showing p-AMPKα and PGC1α protein expression. **d** Quantification of *p*-AMPKα and PGC-1α protein in the TA muscle of obese mice (*n* = 3, 3). **e** Relative mRNA expression of oxidative phosphorylation-related genes in the TA tissue of obese mice (*n* = 6, 6). **f** Relative mRNA expression of fatty acid oxidation-related genes in the TA tissue of obese mice (*n* = 6, 6). Data are expressed as the mean ± standard error of the mean (SEM). *p < 0.05, **p < 0.01 and ***p < 0.001, two-tailed Student *t*-tests (A-D). F/F Follistatin F/F mice, KO FAPs-specific follistatin KO mice, *mRNA* Messenger RNA, *AU* Arbitrary units, *TA* Tibialis anterior

Our previous findings, which highlighted the crucial role of follistatin in regulating muscle homeostasis, further prompted us to investigate its effects on metabolic functions, including mitochondrial biogenesis. These processes are required to meet the energy demands of the body during physical activity. We found that follistatin deletion results in skeletal muscle loss within four weeks, as evidenced by MSTN/activin A expression, which is a well-reported negative regulator of muscle mass. While investigating the effect of follistatin deletion and subsequent pathway activation on mitochondrial biogenesis, the mitochondrial biogenesis was comparable in the TA of NC-fed follistatin KO mice and control mice (Supplementary Fig. S5d, e). Strikingly, we found that mitochondrial biogenesis-related genes such as *Idh1, Ucp2, Ckmt2,* and *Cs* were downregulated in follistatin KO obese mice compared to those in their control littermate, follistatin^f/f^ mice (Fig. 5a). PGC-1α is a master regulator of mitochondrial biogenesis; hence, we assessed the expression of various transcription factors, including *Ppargc-1a, Tfam,* and *Errβ*. *Ppargc-1a* and *Tfam* were significantly downregulated in follistatin KO obese mice compared to that in follistatin^f/f^ control mice, while a decrease in *Errβ* expression was observed, it was not statistically significant (Fig. 5b). PGC-1α is also known to be regulated by AMPKα (Irrcher et al. 2008). Our western blot analysis further confirmed that p-AMPkα as well as PGC-1α protein level in the TA of follistatin KO obese mice was reduced than that in the control littermates (Fig. 5c, d). Furthermore, we showed that the oxidative phosphorylation-related genes, *Ndufb8, Ndufv1, Sdhb,* and *Cox5b,* were also downregulated in the TA muscle of follistatin KO obese mice (Fig. 5e). In addition to this, the expression of genes related to fatty acid oxidation, including *Acox3* and *Acacb,* was reduced in the TA muscle of follistatin KO obese mice (Fig. 5f). Taken together, the observed increase in *p*-SMAD2/3 and FOXO1 activity associated with a decline in p-AMKα results in a reduction of mitochondrial function-related genes in obesity, thereby modulating muscle homeostasis and mass.

### FAPs-derived follistatin has a direct effect on C2C12 cells *in vitro*

**Fig. 6:**
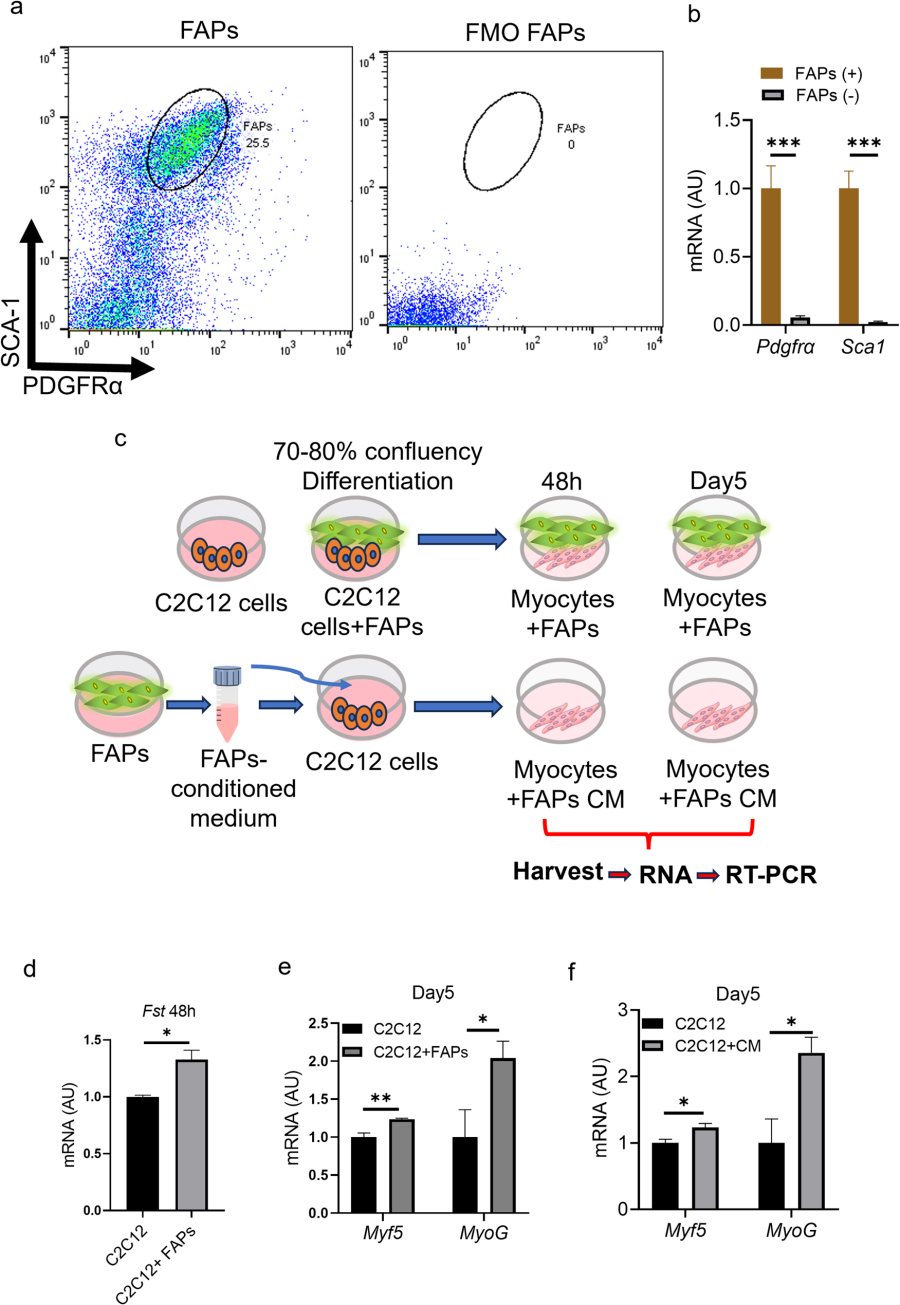
FAPs-derived follistatin has a direct effect on C2C12 cells in vitro (**a**) Representative flow cytometry images of CD45-CD31-PDGFRa+SCA1+ FAPs isolated from the TA and GC muscle of wild-type mice and Fluorescence minus one (FMO) for the justification of the flow cytometry gating strategy. **b** Relative mRNA expression of *Pdgfrα *and *Sca1 *in FACS-sorted FAPs (+) and FAPs (-) cells from the GC of wild-type mice (*n* = 5, 5). **c** Schematic diagram showing the experimental setup. **d** Relative mRNA expression of *Fst*, in C2C12 cells co-cultured with FAPs after 48h (*n* = 3, 4). **e** Relative mRNA expression of *Myf5 *and *MyoG*, in C2C12 cells co-cultured with FAPs after day 5 (*n* = 3, 4). **f** Relative mRNA expression of *Myf5 *and *MyoG *in C2C12 cells treated with CM from FAPs after day 5 (*n* = 3, 4). Data are expressed as the mean ± standard error of the mean (SEM). *p < 0.05, two-tailed Student t-tests (A-D). *FAPs *Fibro-adipogenic progenitors, *mRNA *messenger RNA, *AU *Arbitrary units, *CM *Conditional medium

FAPs are the mesenchymal stem cells that are known to express high levels of follistatin, which promotes myogenesis. Whether FAPs-derived follistatin has a direct effect on the C2C12 cells, we performed ex vivo co-culture by culturing FAPs with C2C12 cells. Initially, we isolated CD45-CD31-PDGFRα + SCA1 + FAPs (+) and CD45-CD31-PDGFRα-SCA1- FAPs (-) cell populations by FACS-sorting (Fig. 6a) (gating strategy to isolate the FAPs is given in supplementary Fig. S1). Our gene expression analysis showed that FAPs (+) cells express higher levels of *PDGFRα* and *Sca1* FAPs genes compared to FAPs (-) cells (Fig. 6b), indicating that FAPs were successfully isolated from the skeletal muscle of wild-type mice by FACS-sorting. We maintained the C2C12 cells and FAPs in growth medium, proliferated and co-cultured them as shown in (Fig. 6c). We investigated the impact of FAPs co-culture at early stages of differentiation at 48 h. We observed increased *Fst* expression in C2C12 cells upon co-culture with FAPs at 48 h of differentiation induction (Fig. 6d). Our gene expression analysis at day 5 showed increased expression of *Myf5* and *MyoG* (Fig. 6e), indicating paracrine regulation. Next, we collected conditioned media (CM) from the FAPs and then treated C2C12 cells. We again observed increased expression of *Myf5* and *MyoG* at day 5 of CM treatment. These data suggest that FAPs might secrete follistatin that promotes differentiation of C2C12 cells to myocytes. Taken together, FAP-derived follistatin is a critical regulator of muscle homeostasis *in vivo* and *in vitro.*

## Discussion

In the current study, we investigated the role of FAPs-derived follistatin in metabolism and skeletal muscle function in obese mice. Follistatin is an extracellular protein that antagonizes many TGF-β superfamily members, including activins (Mather et al. 1997), and prevents these ligands from binding to their type II receptors, thereby neutralizing their activity (Harrison et al. 2005). Inhibition of TGF-β signaling has been shown to restore muscle strength or function (Bogdanovich et al. 2002; Acuña et al. 2014). Here, we focused on examining the role of follistatin in FAPs specifically from obese mice using PDGFRα-GCE; follistatin^f/f^ mice (Nawaz et al. 2022; Bilal et al. 2021). Previous reports showed that the follistatin in circulation correlates positively with fasting blood glucose and HbA1c (Hansen et al. 2013). Liver-specific deletion of follistatin exhibits moderately improved glucose tolerance and increased weight gain (Tao et al. 2023). Here, in the current study, we show that FAPs-specific deletion of follistatin does not alter glucose and insulin tolerance in both lean and obese mice, suggesting a tissue-specific role of follistatin. 

Our data from follistatin KO lean mice showed increased expression of TGF-β family members, including *Mstn* as well as *Inhba* gene encoding activin A, and their subsequent binding receptors, like *Alk4* and *Acvr2b* (Fig. 2a), suggesting that TGF-β signaling is activated, thereby affecting physiological skeletal muscle function. Previously, by using the same mouse model in lean mice, we reported that FAPs-specific deletion of follistatin following acute injury impaired the muscle regeneration process (Nawaz et al. 2022). These outcomes demonstrate that Fst plays an essential role in steady-state conditions and in response to cardiotoxin-induced muscle damage.

In obese mice, we showed that follistatin KO mice displayed reduced exercise performance without affecting glucose tolerance or insulin sensitivity. Previous studies have also emphasized the critical role of follistatin in regulating muscle homeostasis via satellite cell activation (Gilson et al. 2009; Feger et al. 2022) and have shown that it might control exercise performance. Strikingly, our follistatin KO mice exhibited reduced skeletal muscle mass, specifically of the TA and GC muscles, four weeks after deleting follistatin in the muscles, which is indicative of accelerated muscle wasting. Further analysis revealed upregulation of TGF-β superfamily members, including MSTN and activin A (Fig. 2), which negatively regulate muscle mass.

MSTN is a potent negative regulator of muscle mass and myofiber size, affecting muscle growth and development. Mice lacking *Mstn* exhibited robust enhancement in skeletal muscle mass, displaying a phenotype called “double-muscle” across various species (Kambadur et al. 1997; Grisolia et al. 2009; Dilger et al. 2010). Another TGF-β family member, activin A, has also been reported to negatively regulate muscle mass by cooperating with MSTN, thereby dramatically affecting muscle phenotype (Lee et al. 2020). Both MSTN and activin A signal through common type II receptors, particularly ACVR2B and type I ALK4/5 activin receptors, thereby activating the p-SMAD2/3 signaling pathway (Pangas and Woodruff 2000; Namwanje and Brown 2016).

Previous studies have shown that follistatin overexpression in transgenic mice or via the adenovirus induces muscular hypertrophy (Gilson et al. 2009; Feger et al. 2022); this effect was even more than that observed in *Mstn* KO mice (Gilson et al. 2009). Thus, both MSTN and activin A are under the control of follistatin (Feger et al. 2022). Here, we found upregulated MSTN/activin A signaling following *Fst* downregulation in FAPs. Subsequently, we found increased SMAD2/3 phosphorylation in the TA of the follistatin KO mouse model, which may regulate their target genes and induce skeletal muscle loss. Additionally, *FoxO* family genes harbor binding sites for the SMAD2/3 transcription factors, which regulate the expression of muscle atrophy-related genes (Gumucio and Mendias 2013). The activation of SMAD2/3 leads to degradation of muscle proteins, including Atrogin1 and MuRF1, via FOXO1 (Goodman et al. 2013; Han et al. 2013). FOXO1 is also controlled by the SMAD2/3 canonical signaling pathway, which dephosphorylates FOXO1, mediates its nuclear translocation, and thereby induces atrophy-related genes (Goodman et al. 2013). Consistent with this increased p-SMAD2/3, western blotting and gene expression analysis showed that FoxO1 activity was enhanced in the TA of follistatin KO mice compared to that in control follistatin^*f*/f^ mice (Fig. 4c-e). In agreement with this, another study demonstrated that mice lacking FoxO1,3,4 (triple KO mice) are protected from muscle weakness and atrophy (Milan et al. 2015). Subsequently, muscle atrophy-related genes, including *Atrogin1, MuRF1, Bnip3* and *Klf15,* were also upregulated following MSTN:activin A/p-SMAD2/3/FoxO1 pathway induction in follistatin KO mice. In contrast, a previous report showed that MSTN, independent of SMAD3 or NF-κB, can upregulate the expression of *MuRF1,* thereby inducing muscle degradation (Sriram et al. 2014). A study conducted on chick embryonic myotubes demonstrated that MSTN could induce Atrogin1 expression by inducing SMAD2 phosphorylation independent of FOXO1 (Saneyasu et al. 2019). Deletion of *MuRF1* attenuates muscle atrophy in a mouse model of denervation-induced muscle atrophy (Bodine et al. 2001). Based on our results and the observations of previous studies, we suggest that FAPs-specific follistatin is critical for muscle growth and homeostasis and that it acts by blocking TGF-β signaling (MSTN: Activin A/p-SMAD2/3/FoxO1) in obese mice, thereby playing an important role in physical activity.

Cyclin-dependent kinases (Cdks) and CKIs regulate cell cycle progression in mammals. CKIs comprise two families, Ink and Kip/Cip, where p15^ink^, p16^ink^, p18^ink^, and p19^ink^ belong to the Ink family and p121^cip^, p27^kip^, and p57^kip^ belong to the Kip/Cip family. CKIs play a fundamental role in sustaining growth arrest and eliminating cellular differentiation (Johnson and Walker 1999). p27^kip^ regulates the G1 phase of cell cycle progression (Phelps and Xiong 1998; Toyoshima and Hunter 1994), and mice lacking p27^kip^ show enhanced growth due to hyperplasia (Kiyokawa et al. 1996; Nakayama et al. 1996). Two decades ago, a study showed that cultured muscle precursor cells treated with MSTN exhibited increased p21 expression. A few years later, Lin et al. reported (Lin et al. 2003) that mice lacking p27^kip^ showed growth enhancement, including an increase in skeletal muscle mass, with reduced MSTN levels in skeletal muscles. p27 is mainly studied in a developmental context, and its role in mature muscle is underexplored. MSTN and p27 might be closely associated and regulate muscle homeostasis. Therefore, we investigated p27^kip^ signaling in a follistatin KO mouse model. Accordingly, follistatin KO mice exhibited increased *p27* mRNA levels, while the mRNA levels of other CKIs, including *p16, p21* and *p57,* were also increased. IHC analysis also showed an increase in the p27 signal in the TA of follistatin KO mice (Fig. 3). The signal of p27 was mainly enriched in the non-myofiber cells, which might be satellite cells or FAPs. The CKIs (p16, p21, p27, and p57) lies downstream of TGF-β, and may regulate these effects by regulating satellite cell function or FAPs to fibrosis. Recently, in the acute injury mouse model, we reported that depletion of CD206^+^ M2-like macrophages in the skeletal muscle enhances muscle regeneration due to the reduction of TGF-β1 and enhanced FAPs-derived follistatin (Nawaz et al. 2022). Furthermore, the p27 signal was also reduced along with the reduction of TGF-β1 in the CD206^+^ M2-like macrophage-depleted mice (Nawaz et al. 2022). In addition to this, knockdown of CD206-specific TGF-β1 also resulted in increased follistatin and enhanced myogenesis (Nawaz et al. 2022). These findings suggest that follistatin and TGF-β signaling, along with their downstream molecules, including p27, are closely interlinked in regulating muscle homeostasis.

Mitochondria generate most of the chemical energy in the form of ATP required to power cellular metabolic processes. They are also involved in several other important cellular functions, including the production of reactive oxygen species, apoptosis, and programmed cell death (Brand et al. 2013), and play a critical role in maintaining skeletal muscle health (Swalsingh et al. 2022). In contrast, mitochondrial failure triggers catabolic pathways, leading to skeletal muscle atrophy. (Romanello and Sandri 2016). In follistatin KO mice, reduced exercise performance, skeletal muscle loss, increased muscle degradation, and atrophy prompted us to investigate mitochondrial biogenesis, oxidative phosphorylation, and fatty acid oxidation-related pathways. As expected, mitochondrial biogenesis, oxidative phosphorylation, and fatty acid oxidation were downregulated in the muscles of follistatin KO mice compared to those in control mice, as shown in Fig. 5. PGC-1α*,* the master regulator of mitochondrial biogenesis, was mainly downregulated. A previous report has shown that SMAD3 suppresses the activity of PGC-1α in skeletal muscle cells, thereby negatively regulating mitochondrial biogenesis (Tiano et al. 2015). Furthermore, SMAD3 has binding sites on the PGC-1α promoter in 3T3-L1 adipocytes as well as in C2C12 myofibroblasts, which suppress its expression (Tiano et al. 2015; Yadav et al. 2011). Taken together, the observed increase in p-SMAD2/3 and FOXO1 coincides with markers of reduced mitochondrial function; however, further studies, including targeted rescue or inhibition experiments, are required to determine whether these pathways play a causal role in mitigating metabolic dysfunction in this model.

This study has a few limitations: first, we investigated the effect of short-term (just four weeks) follistatin deletion on muscle phenotype in obese mice. Second, we focused on only skeletal muscle and did not investigate the effect of follistatin deletion in adipose tissue, where adipocyte progenitors are the major source of follistatin. Thirdly, we did not investigate the role of follistatin deletion in myogenesis following an acute muscle injury in obese mice. We analyzed the physiological effect of follistatin deletion in FAPs in response to exercise in obesity; the role of FAPs-derived follistatin in adaptation to exercise in NC and HFD-fed mice needs to be examined. Future studies are warranted to investigate the effect of long-term follistatin deletion on muscle, the aging process, and adaptation to exercise. We are also interested in examining the role of follistatin in adipose tissue and liver in future research.

## Conclusion

This study reveals the role of FAPs-derived follistatin in the skeletal muscles of HFD-fed obese mice. Our findings revealed that deletion of the follistatin in FAPs reduced exercise ability and disturbed skeletal homeostasis via the MSTN:activin A/p-SMAD2/3 signaling cascade, thereby regulating muscle mass and homeostasis. Furthermore, follistatin KO mice showed reduced muscle mass, increased muscle proteolysis, and muscle atrophy. The transcripts of various metabolic pathways, including mitochondrial biogenesis, oxidative phosphorylation, and fatty acid oxidation, were also reduced in the skeletal muscles of follistatin KO mice. Together, these results implied that follistatin protects mice by preserving the metabolic health of skeletal muscles and maintaining skeletal muscle function during HFD intervention, which may lessen the consequences of diet-induced obesity, such as sarcopenia.

## Supplementary Information

Supplementary Material 1

Supplementary Material 2

## Data Availability

No datasets were generated or analysed during the current study.

## Abbreviations

BAT: Brown adipose tissue
BW: Body weight
ETT: Exercise tolerance test
eWAT: Epididymal white adipose tissue
FAPs: Fibro-adipogenic progenitors
FST: Follistatin
Follistatin^f/f^: Follistatin^fl^^ox/^^fl^^ox^
GST: Grip strength test
HFD: High-fat diet
Ip-GTT: Intraperitoneal glucose tolerance test
Ip-ITT: Intraperitoneal insulin tolerance test
iWAT: Inguinal white adipose tissue
MSTN: Myostatin
NC: Normal chow
TAM: Tamoxifen
CKIs: Cyclin-dependent kinase inhibitors
IHC: Immunohistochemistry
WAT: White adipose tissue
SEM: Standard error of the mean
FBS: Fetal bovine serum
DMEM: Dulbecco’s modified Eagle’s medium
CM: Conditional medium
