# Supplementary material for: Deletion of fibro-adipogenic progenitors-specific follistatin impairs muscle function and accelerates skeletal muscle atrophy in obese mice
Source: Mol Med. 2025 Nov 21;31:340. doi: 10.1186/s10020-025-01393-1 (PMC12752164; doi:10.1186/s10020-025-01393-1)

Supplementary Figure. 1

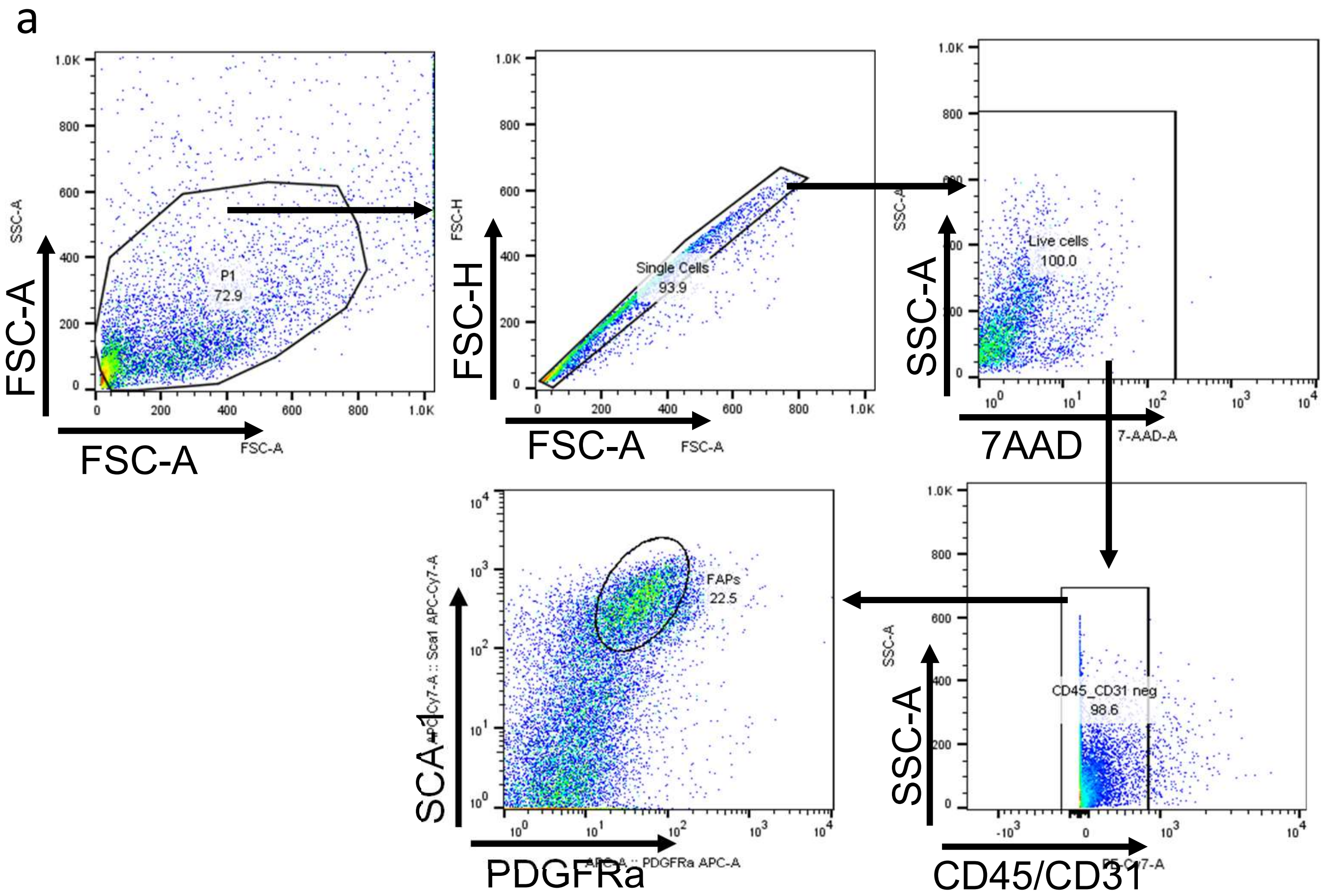

Fig. S1. Flow cytometric analysis of GC muscle (a) Flow cytometric gating strategy for the analysis of FAPs

Supplementary Figure. 2

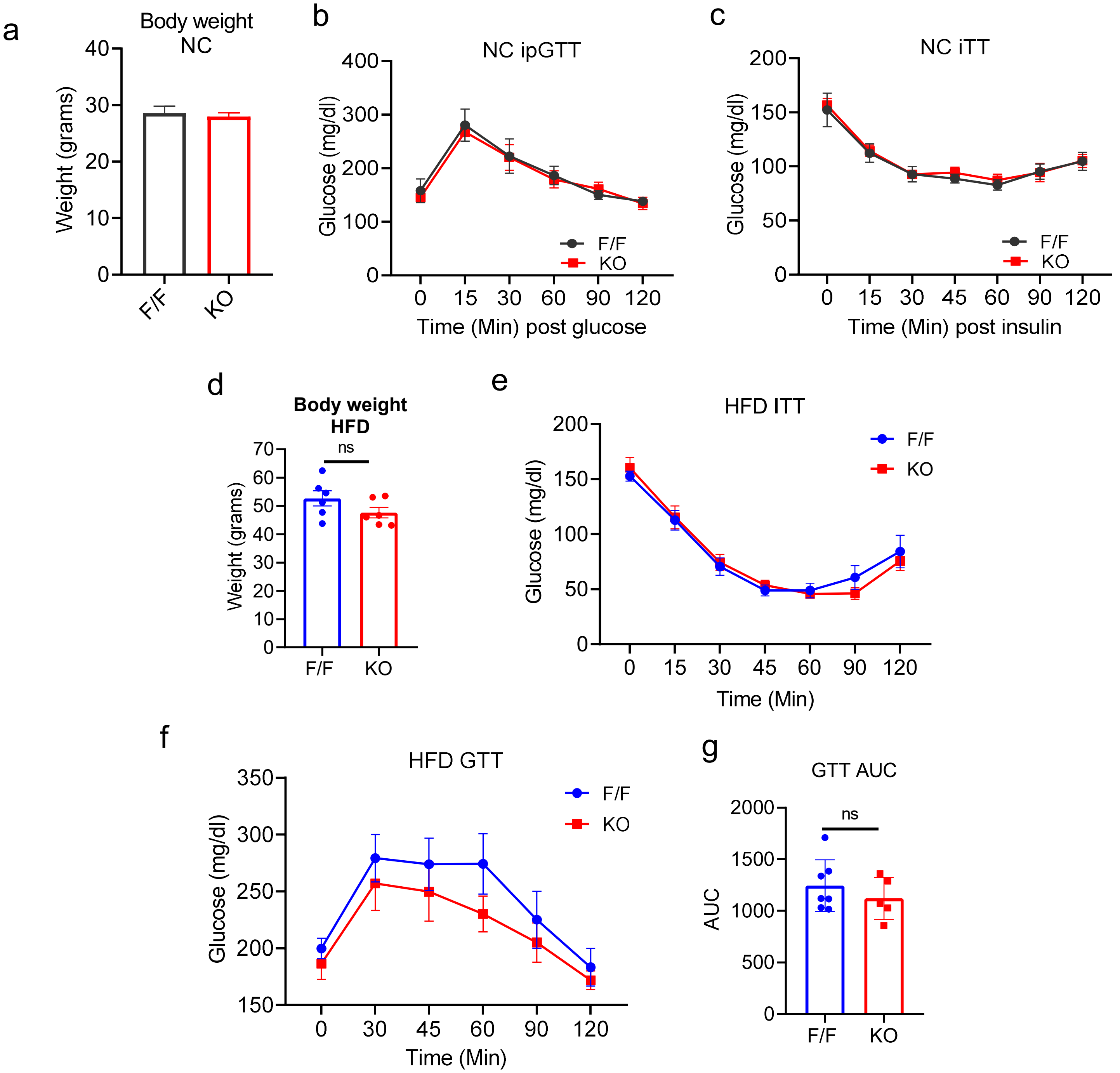

Fig. S2. Deletion of *Fst* in FAPs does not alter metabolic homeostasis in NC and HFD-fed mice (a) Body weights of NC-fed mice. (n = 4, 4). **(b)** Insulin tolerance test (ITT) in mice after 20 weeks of NC feeding (n = 4, 5). **(c)** Glucose tolerance test (GTT) in mice after 20 weeks of NC feeding (n = 4, 5). **(d)** Body weights of mice after 20 weeks of HFD feeding (n = 7, 5). **(e)** Insulin tolerance test (ITT) in mice after 19 weeks of HFD feeding (n = 7, 5). **(f)** Glucose tolerance test (GTT) **(g)** Area under curve (AUC) in mice after 19 weeks of HFD feeding (n = 7, 5).

(e) Relative mRNA expression of muscle protein degradation-related genes, FoxO1, *Atrogin1, MuRF1,* and *Bnip3,* in the TA tissue of lean mice (n = 3, 4).

Supplementary Figure. 3

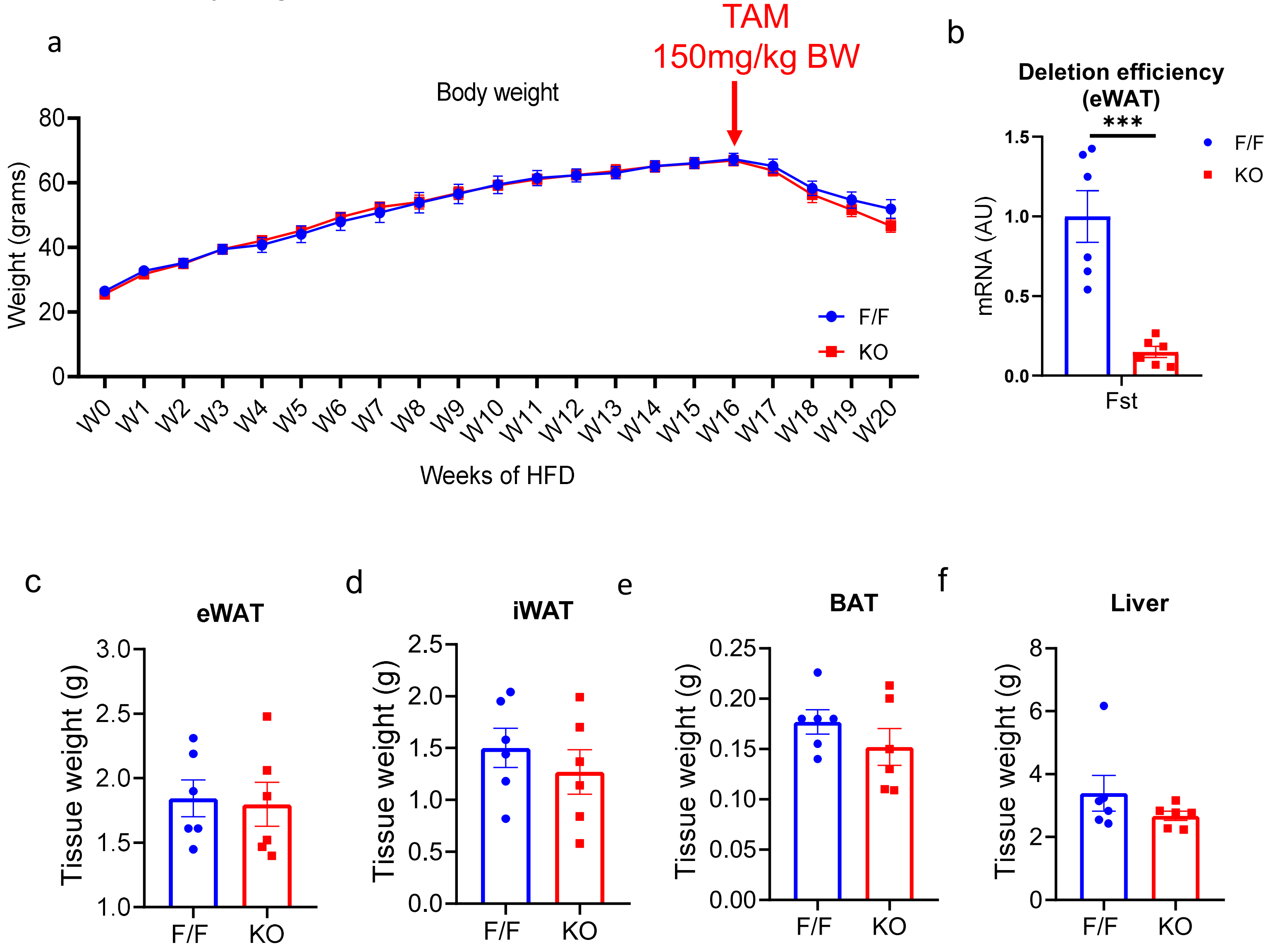

Fig. S3: Body weight and tissue weights of sacrificed mice fed HFD for 20 weeks.
(a) Time course of body weight changes of f/f and KO mice after 20 weeks of HFD feeding. (n = 6, 6). **(b)** Relative mRNA expression of *Fst* in the eWAT tissue of obese mice (n = 6, 6). **(b-e)** Skeletal muscle weight of TA, GC, and soleus after 20 weeks of HFD feeding (n = 6, 6).

Data are expressed as the mean ± standard error of the mean (SEM). ***p < 0.001, F/F: Follistatin F/F mice; KO: PDGFRα-specific follistatin KO mice; mRNA messenger RNA; AU: arbitrary units.

Supplementary Figure. 4

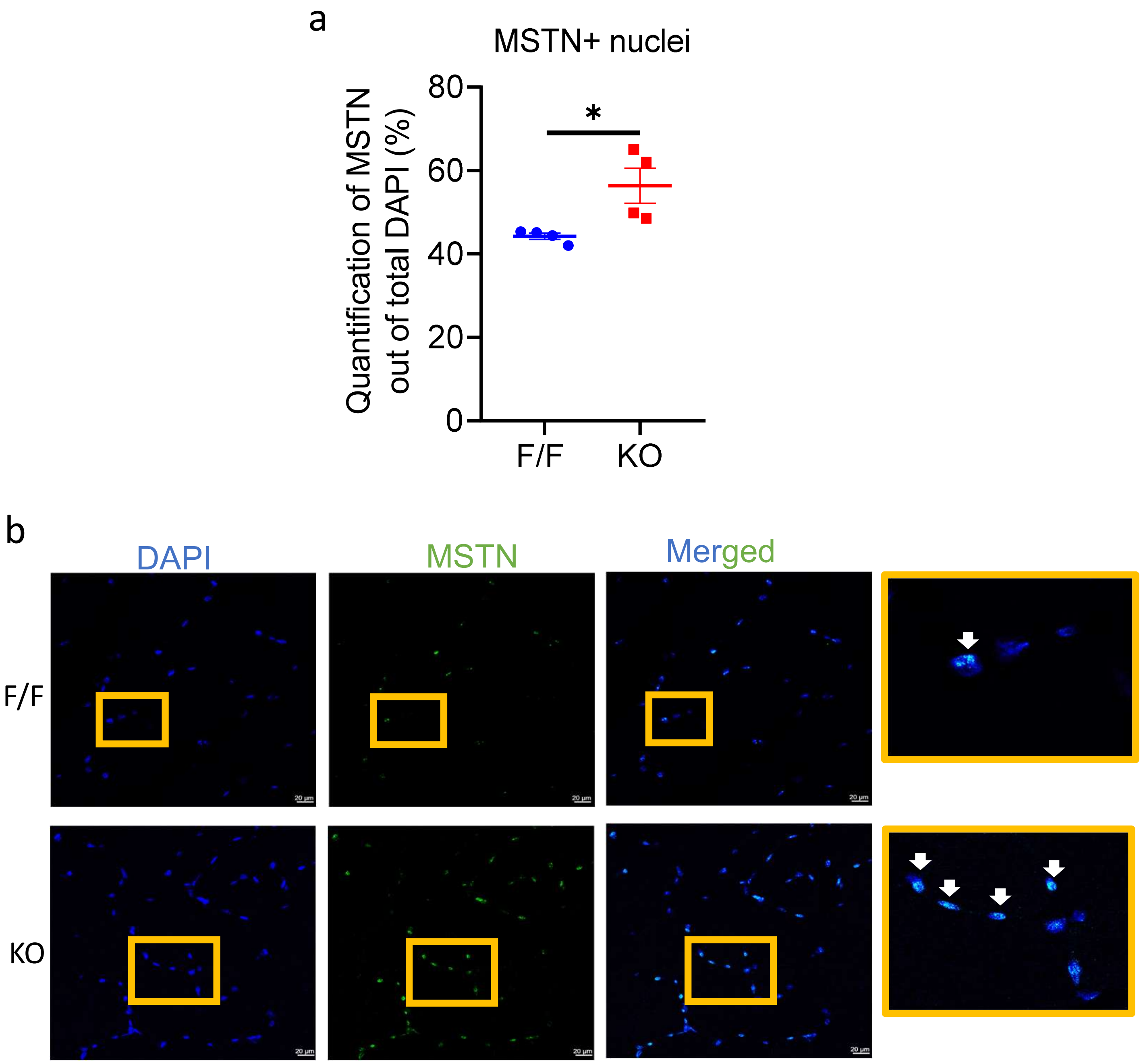

Fig. S4: MSTN signal is increased in follistatin KO mice.
(a) Quantification of MSTN+ nuclei out of total DAPI-stained nuclei in the TA from obese mice (n = 4 each). (b) Representative confocal images of TA stained with anti-MSTN (green) and DAPI (blue) in obese mice (n = 4 mice/group/experiment; scale bar, 20 μm).

Supplementary Figure. 5

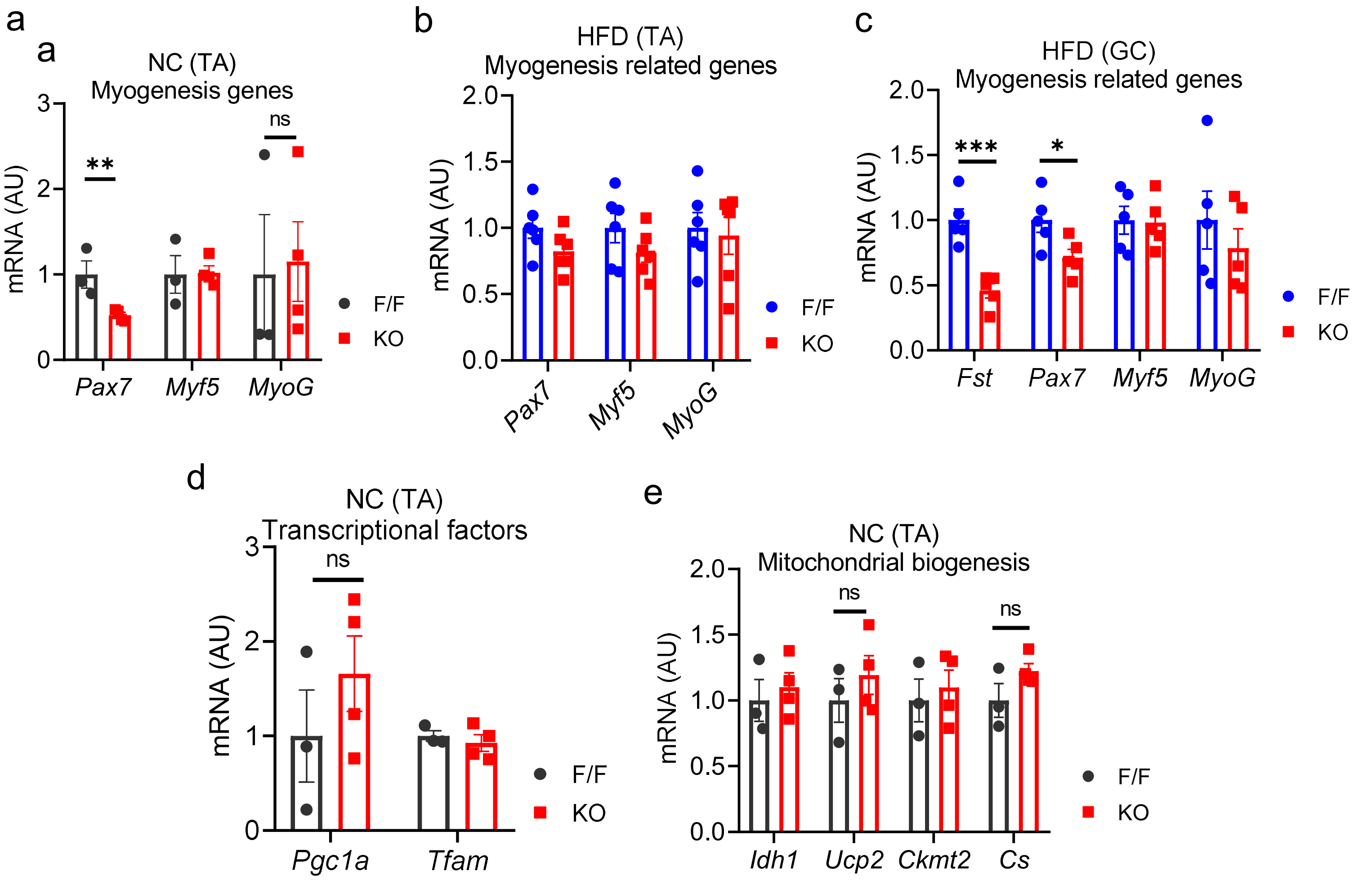

Fig. S5: Pax7 is downregulated in follistatin KO mice without alteration in other myogenic genes and mitochondrial biogenesis is comparable in NC-fed mice.

(a) Relative mRNA expression of myogenesis-related genes, *Pax7, Myf5* and *MyoG,* in the TA muscle of lean mice (n = 3, 4). **(b)** Relative mRNA expression of myogenesis-related genes, *Pax7, Myf5* and *MyoG,* in the TA muscle of obese mice (n = 6, 6). **(c)** Relative mRNA expression of myogenesis-related genes, *Pax7, Myf5* and *MyoG,* in the GC muscle of obese mice (n = 6, 6). **(d)** Relative mRNA expression of transcription factors, *Pgc1a,* and *Tfam,* which regulated mitochondrial biogenesis in the TA tissue of lean mice (n = 3, 4). **(e)** Relative mRNA expression of mitochondrial biogenesis-related genes, *Idh1, Ucp2, Ckmt2* and *Cs,* in the TA tissue of lean mice (n = 3, 4).

Data are expressed as the mean ± standard error of the mean (SEM). ***p < 0.001, F/F: Follistatin F/F mice; KO: PDGFRα-specific follistatin KO mice; mRNA messenger RNA; AU: arbitrary units.

Supplementary Figure. 6

GDF8/MSTN

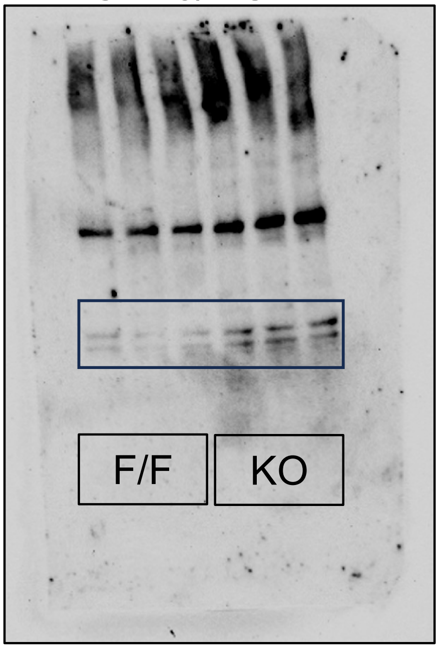

α-Tubulin

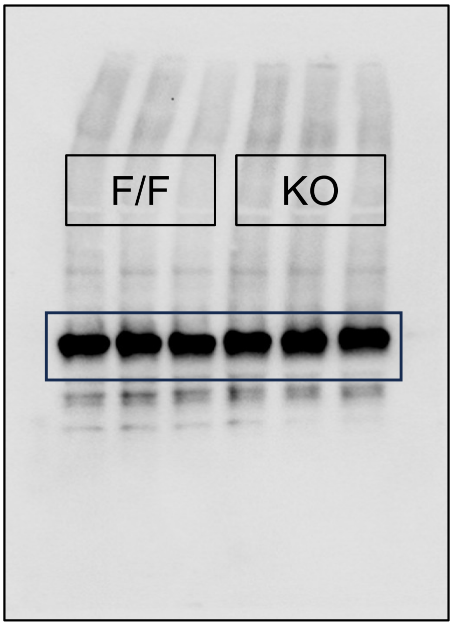

Supplementary Figure. 7

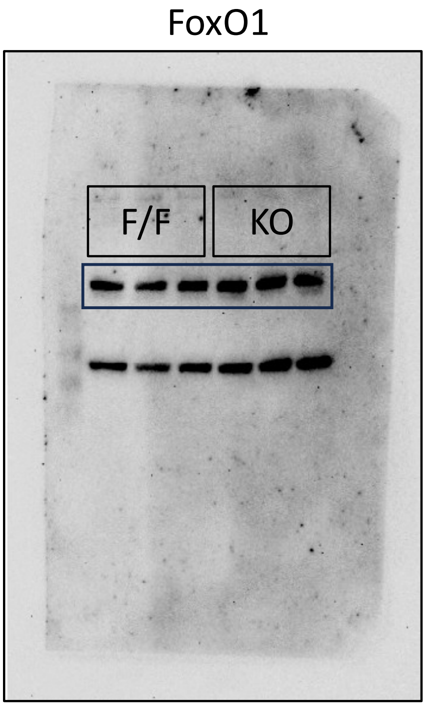

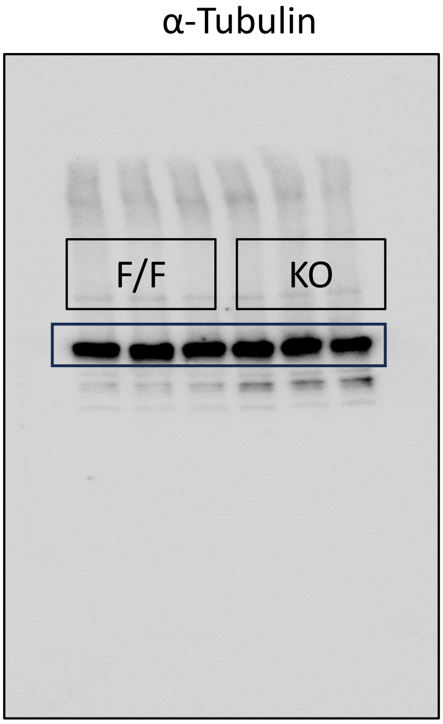

Supplementary Figure. 8

p-AMPKa

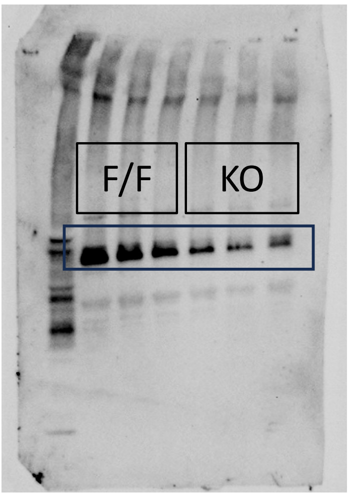

AMPKα

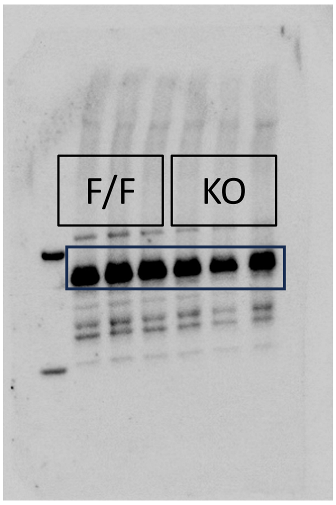

Supplementary Figure. 9

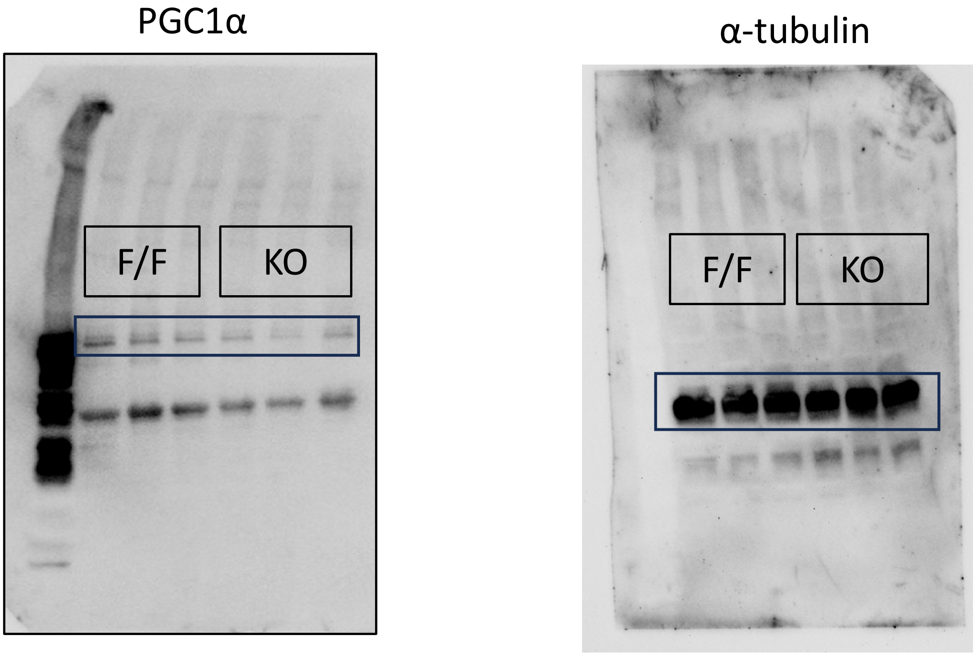

Supplement: Supplementary file 1 — Supplementary Material 1 [file 10020_2025_1393_MOESM1_ESM.pdf]
