# Supplementary material for: Deletion of fibro-adipogenic progenitors-specific follistatin impairs muscle function and accelerates skeletal muscle atrophy in obese mice
Source: Mol Med. 2025 Nov 21;31:340. doi: 10.1186/s10020-025-01393-1 (PMC12752164; doi:10.1186/s10020-025-01393-1)
Supplement: Supplementary file 2 — Supplementary Material 2 [file 10020_2025_1393_MOESM2_ESM.docx]

| **Supplementary table 1. Key resources** |  |  |
| --- | --- | --- |
| **Reagent Or Resourse** | **Source** | **Identifier** |
|  |  | **(Catalog #)** |
| **Antibodies** | | |
| FoxO1 (L27) Antibody | Cell Signaling Technology | 9454 |
| P27^kip1^ (SX53G8.5) Rabbit mAb | Cell Signaling Technology | 3698 |
| P-Smad2/3 Rabbit mAb | Cell Signaling Technology | 8828S |
| Rat monoclonal anti-Laminin alpha2 | Santa Cruz | Sc-59854 |
| GDF8/MSTN Polyclonal Antibody | Bioss | bs-1288R |
| P-AMPKα Rabbit mAb | Cell Signaling Technology | 2535P |
| AMPKα Rabbit mAb | Cell Signaling Technology | 5831P |
| Goat anti-Rabbit IgG (H+L) Highly Cross-Adsorbed, Alexa Fluor Plus 488 | Invitrogen | A32731 |
| Anti-Rat IgG (H+L) Highly Cross-Adsorbed, Alexa Fluor Plus 488 | Cell Signaling Technology | 4416S |
| Goat anti-Rabbit IgG (H+L) Highly Cross-Adsorbed, Alexa Fluor Plus 568 | Invitrogen | A11036 |
|  |  | Lot# AM70730A |
| Takara Primescript RNA Kit | (Shiga, Japan) | RR036A |
| The ECL^TM^ western blot detection reagent | GE Healthcare | RPN2232 |
| **Chemicals** | | |
| Collagenase | Sigma-Aldrich | C6885 |
| Tamoxifen | Sigma-Aldrich | T5648 |
| D-Glucose-6,6-D2 | Santa Cruz Biotechnology | Sc-257287A |
| Direct PCR (Tail) Lysing Solution | Viagen (Los Angeles, CA) | N/A |
| Bovine serum albumin | Sigma-Aldrich | A8022-100G |
| BD Pharm lyse lysing buffer | BD Biosciences | 555899 |
| PBS(-) | Nacalai tesque | Code 14249-24 |
| DAKO real antibody diluent | DAKO | Ref# S2022 |
| Normal goat serum | Vector Laboratories | Ref# S-1000 |
| Fetal bovine serum (FBS) | Gibco | Ref# 10437-028 |
| Ethanol (99.5%) | Fujifilm WAKO Chemicals. | 057-00456 |
| Dulbecco’s modified Eagle’s medium (DMEM) | Nacalai Tesque | Code 08459-64 |
| 0.1% Gelatin solution in water | Stem cell technologies | 07903 |
| 4% Paraformaldehyde phosphate buffer solution | Fujifilm WAKO Chemicals. | 163-20145 |
| Direct PCR (Tail) | Viagen Biotech Incorporation | 102-T |
| Proteinase K, recombinant PCR | Roche Diagnostics | 03115828001 |
| grade |  | Lot# 48602000 |
| Ethidium bromide | Invitrogen | 15585-011 |
| Agarose S gel | Nippon Gene | 312-01193 |
| New hematoxylin solution | Muto Pure chemicals Co, | Lot# 131209 |
|  | limited |  |
| New eosin solution | Muto Pure chemicals Co, | Lot# 131028 |
|  | limited |  |
| β-Mercaptoethanol | Gibco | 21985-023 |
| **Experimental Models: Organisms/Strains** | | |
| Fst^flox/flox^ mice | Nawaz, A. et all 2022 | N/A |
| Pdgfrα-CreERT2-Egfp mice | Horikawa, S. et al., 2015 | N/A |
| **Software and Algorithms** | | |
| ImageJ Software 11.53a | National Institute of Health, | online |
|  | USA |  |
| GraphPad Prism | Version 9 |  |
| **Other** | | |
| Normal chow (NC) diet | CLEA, Japan | CE-2 |
| HFD (60% KCAL Fat) | Research Diets | D12492 |
|  |  | Lot# 20120201 |

**Supplementary table 2. Primer’s list used for QPCR**

| Primer　Name | Sequence |
| --- | --- |
| *Acacb* | Forward: TGTTCTCGGCCTCTCTTCAC Reverse: GAGGCTGCATTGAACACAAG |
| *Acox1* | Forward: GGAATTTGGCATCGCAGACC Reverse: ACATGCCCAAGTGAAGGTCC |
| *Acox3* | Forward: GCTCGGTAGGCACTAAGAGG Reverse: CTTCTGAGAAACGGGGACAA |
| *Acvr2b* | Forward: GTTCATTGCTGCCGAGAAAC Reverse: GATGTTCCCCTTGAGGTAATCC |
| *Alk4 (acvr1b)* | Forward: TGCTTGAGCTTTCTGTGCAT Reverse: GAGAAGCAGCAGCACTCAGA |
| *Atp5c1* | Forward: CCAGGAGACTGAAGTCCATCA Reverse: AGAACCTGTCCCATACACTCG |
| *Atrogin-1* | Forward: GAGACCATTCTACACTGGCAGCA Reverse: GTCACTCAGCCTCTGCATGATGT |
| *Beta-actin* | Forward: GCCGGGACCTGACAGACTAC Reverse: AACCGCTCGTTGCCAATAGT |
| *Bnip3* | Forward: ATTGCCATTGCTGAAGTGC  Reverse: CCCTGCTACCTCTCGGTGAC |
| *Ckmt2* | Forward: TTCGGCCAGTTAGCAACTTT  Reverse: TTACACTTCTTGGCTGTGTGC |
| *Cox5b* | Forward: GGAAGACCCTAATCTAGTCCCG Reverse: GTTGGGGCATCGCTGACTC |
| *Cpt1a* | Forward: TTGGAAGTCTCCCTCCTTCA Reverse: GCCCATGTTGTACAGCTTCC |
| *Cs* | Forward: AAGGACGAGGCAGGATGAG Reverse: TGCAGCTGTAGCTCTCTCCC |
| *Errb* | Forward: TGAAGGAGCCGCAACTAGAG Reverse: GCTGGAACACCTGAGGGTAA |
| *Foxo1* | Fw;GGGTTAGTGAGCAGGTTACAC Rev;TCCAATGGCACAGTCCTTATC |
| *Fst (follistatin)* | Forward: GACAATGCCACATACGCCAG Reverse: GTTTCTTCCGAGATGGAGTTGC |
| *Fstl1* | Forward: GCCAGCTCCACAAAACACAT  Reverse: GAGCACGATGTGGAAACGAT |
| *Inhba* | Forward: ATCATCACCTTTGCCGAGTC  Reverse: GTTAGCCTTGGGGACTTTCAG |
| *Idh1* | Forward: CTCTGCAGCATCTTTGGTGA Reverse: TGACACGAATCATTTGGGAA |
| *MuRF1* | Forward: ACCTGCTGGTGGAAAACATCATT Reverse: AGGAGCAAGTAGGCACCTCACAC |
| *Myf5* | Forward: GACGGCATGCCTGAATGTAAC Reverse: GCTGGACAAGCAATCCAAGC |
| *Myog* | Forward: GTGAATGCAACTCCCACAGC Reverse: CGCGAGCAAATGATCTCCTG |
| *Ndufb8* | Forward: CCCGCTCCAGGTACAGATTA Reverse: GCTTTGTGGCTTTCATGGTT |
| *Ndufv1* | Forward: GTGCGGGTATCTGTGCGTT Reverse: GGTTGGTAAAGATCCGGTCTTC |
| *Ndusf2* | Forward: TCGTGCTGGAACTGAGTGGA Reverse: GGCCTGTTCATTACACATCATGG |
| *p16* | Forward: GAACTCTTTCGGTCGTACCC Reverse: CGAATCTGCACCGTAGTTGA |
| *p21* | Forward: GACATCTCAGGGCCGAAAAC Reverse: CGGCGCTTGGAGTGATAGAA |
| *p27* | Forward: AGGAGAGCCAGGATGTCAGC Reverse: CAGAGTTTGCCTGAGACCCAA |
| *p57* | Forward: GGAGCAGGACGAGAATCAAG Reverse: GAAGAAGTCGTTCGCATTGG |
| *Pax7* | Forward: CGGGTTCTGATTCCACATCT Reverse: CGACGAGGAAGGAGACAAGA |
| *Ppargc1a* | Forward: CCCTGCCATTGTTAAGACC Reverse: TGCTGCTGTTCCTGTTTTC |
| *Ppara* | Forward: TTTTCCGAAGAACCATCCGATT Reverse: ATGGCATTGTGAGACATCCCC |
| *Sdhb* | Forward: TCTGGGTCCCATCGGTAAAT Reverse: GCCGTTCTCGGCAGAGTC |
| *Tfam* | Forward: CAGGAGGCAAAGGATGATTC Reverse: CCAAGACTTCATTTCATTGTCG |
| *Tgfb1* | Forward: AAGTTGGCATGGTAGCCCTT Reverse: GCCCTGGATACCAACTATTGC |
| *Ucp2* | Forward: TGGAAAATCGAGGGGATCGG Reverse: GGAGAAACGGGGACCTTCAA |
| *Tf2b* | Forward: TGGAGATTTGTCCACCATGA Reverse: GAATTGCCAAACTCATCAAAACT |

**Lead contact**

Further information and requests for resources and reagents should be directed to and will be fulfilled by the Lead Contact, Kazuyuki Tobe ([tobe@med.u-toyama.ac.jp](mailto:tobe@med.u-toyama.ac.jp))
